# Stomach Microbiome Simplification of a Coral Reef Fish at Its Novel Cold‐Range Edge Under Climate Change

**DOI:** 10.1111/mec.17704

**Published:** 2025-02-22

**Authors:** Chloe Hayes, Angus Mitchell, Roger Huerlimann, Jeffrey Jolly, Chengze Li, David J. Booth, Timothy Ravasi, Ivan Nagelkerken

**Affiliations:** ^1^ Southern Seas Ecology Laboratories, School of Biological Sciences The University of Adelaide Adelaide South Australia Australia; ^2^ Marine Climate Change Unit Okinawa Institute of Science and Technology Graduate University Onna‐son Okinawa Japan; ^3^ School of the Life Sciences University of Technology Sydney Ultimo New South Wales Australia; ^4^ Australian Research Council Centre of Excellence for Coral Reef Studies James Cook University Townsville Queensland Australia

**Keywords:** climate change, coral reef fishes, ecology, microbiome, range‐extension

## Abstract

Climate‐driven range extensions of animals into higher latitudes are often facilitated by phenotypic plasticity. Modifications to habitat preference, behaviour and diet can increase the persistence of range‐extending species in novel high‐latitude ecosystems. These strategies may be influenced by changes in their gut and stomach microbial communities that are critical to host fitness and potentially adaptive plasticity. Yet, it remains unknown if the gut and stomach microbiome of range‐extending species is plastic in their novel ranges to help facilitate these modifications. Here, we categorised stomach microbiome communities of a prevalent range‐extending coral reef fish along a 2000‐km latitudinal gradient in a global warming hotspot, extending from their tropical core range to their temperate cold range edge. At their cold range edge, the coral reef fish's stomach microbiome showed a 59% decrease in bacterial diversity and a 164% increase in the relative abundance of opportunistic bacteria (*Vibrio*) compared to their core range. Microbiome diversity was unaffected by fish body size, water temperature, physiology (cellular defence and damage) and habitat type (turf, barren, oyster, kelp and coral) across their range. The observed shifts in microbiome composition suggest dysbiosis and low plasticity of tropical range‐extending fishes to novel environmental conditions (e.g., temperate prey and lower seawater temperature) at their novel range edges, which may increase their susceptibility to disease in temperate ecosystems. We conclude that fishes extending their ranges to higher latitudes under ocean warming can experience a simplification (i.e., reduced diversity) of their stomach microbiome, which could restrict their current rate of range extensions or establishment in temperate ecosystems.

## 1 Introduction

Anthropogenic warming has facilitated the global redistribution of marine and terrestrial species (Parmesan and Yohe 2003; Chen et al. 2011; Pecl et al. 2017). Climate‐driven species redistributions have already altered species interactions and entire ecosystem functioning (Pecl et al. 2017). Marine species are shifting their distributions poleward at a faster rate than terrestrial species (Chen et al. 2011; Burrows et al. 2011; Poloczanska et al. 2013). Poleward range shifts can act as a mechanism to escape thermally stressful conditions at lower latitudes or allow species to inhabit previously inaccessible higher latitudes (Poloczanska et al. 2016). Animals range shifting into high‐latitude environments often modify their diet (Kingsbury et al. 2019; Monaco et al. 2020), habitat preference (Hayes et al. 2024), behaviour (Coni, Booth, and Ferreira et al. 2021), physiology (Mitchell et al. 2023a) and/or morphology (Smith et al. 2016) to enhance their performance in novel ecosystems. However, a fundamentally overlooked response to their range‐shift success is changes in host‐specific microbiomes—the bacterial communities harbouring the internal and external surfaces of organisms (Wilkins et al. 2019).

Microbial communities shape host physiology (Gould et al. 2018), immunity (Gerardo et al. 2020), behaviour (Ezenwa et al. 2012) and metabolism (Dvergedal et al. 2020) and can respond to environmental change faster than their host. Under rapid environmental change, shifts in microbial communities can promote acclimatisation and genetic adaptation (Alberdi et al. 2016; Webster and Reusch 2017; Peterson et al. 2023). Adaptive responses are apparent when microbial communities show a high degree of plasticity in response to environmental change, which can benefit host resilience or adaptation (Alberdi et al. 2016). Species fitness in changing environments can be mediated by host‐associated microbial communities (Pinnow et al. 2023), whereby beneficial microbial communities can enhance thermal tolerance (Jarmillo and Castañeda 2021) and modulate host pathogenic immunity (Fleischer et al. 2022). Hence, shifts in host microbial communities could indirectly mediate host resilience or vulnerability to changing environments. Yet, whether mutualistic relationships between host fitness and host‐associated microbial communities are plastic in animals exposed to novel or changing climatic conditions remains largely unknown.

Microbiome dysbiosis arises when there is an imbalance or shift in the host's natural microbial composition (Petersen and Round 2014). When dysbiosis occurs, the mutually beneficial interaction between the host and their microbiome community is disrupted, leading to a reduction in microbiome diversity and an increase in pathogenic bacteria (Petersen and Round 2014). Across both marine and terrestrial taxa, previous research has shown that increased temperature can alter the host's microbiome composition (Bestion et al. 2017; Watson et al. 2019; Scanes et al. 2021; Moore et al. 2024), which can promote dysbiosis (Greenspan et al. 2020; Suzzi et al. 2023), decreased fitness (Steiner et al. 2022; Risely et al. 2023) and increased susceptibility to disease (Brown et al. 2012) in animals. Thus, future ocean warming may alter microbiome compositions across a wide range of animal taxa, which may disadvantage species fitness under future climate change. Climate‐driven shifts in microbiome communities may facilitate adaptive plasticity, providing benefits to the host adjusting to novel environmental challenges, shaping their overall adaptation to the environment (Kolodny and Schulenburg 2020). While the response between host fitness and host‐associated microbiome communities is well understood in mammals (Suzuki 2017) and other vertebrates (Ley et al. 2008), including fishes in aquaculture settings (Infante‐Villamil et al. 2020), there is still limited research on how fishes that are extending their ranges to higher latitudes under ocean warming experience shifts in their stomach microbiome composition. Most microbiome studies on fish use controlled experimental designs to understand the relationship between fish and their microbiome under climate change. However, these studies may not capture the ecological complexities of natural ecosystems, whereby species are challenged by novel species interactions (Smith et al. 2018; Mitchell et al. 2022), resource competition (Nagelkerken and Munday 2016; Coni, Booth, and Nagelkerken 2021) and habitat degradation/loss (Stuart‐Smith et al. 2021; Coni, Nagelkerken, and Ferreira et al. 2021) under climate change. This emphasises the need for a more comprehensive understanding of how host–microbiome interactions respond to climate change in natural complex ecological settings.

Coral reef fishes contribute to one of the most diverse assemblages of vertebrates globally and are considered increasingly vulnerable to environmental change (Comte and Olden 2017). Climate change has already increased the poleward dispersal of coral reef fishes into subtropical and temperate ecosystems (Booth et al. 2011; Vergés et al. 2014), which has disrupted temperate ecosystem functionality (Nakamura et al. 2013; Vergés et al. 2016) and generated novel species interactions between local and range‐extending species (Smith et al. 2018). There has been substantial focus on what facilitates the poleward movement of coral reef fishes (e.g., increased ocean temperatures, strengthening of boundary currents and species traits; Booth et al. 2007, 2018; García Molinos et al. 2022) and the outcome of their range shifts to temperate ecosystems (e.g., novel species interactions and resource competition, Smith et al. 2018; Coni, Nagelkerken, and Ferreira et al. 2021). Despite this, how fish microbiome diversity and functioning may facilitate or limit their range shifts into novel environments remains relatively unknown (but see Jones et al. 2018). The microbiome of fishes can shape their physiology and ecology (Clements et al. 2014) and correlate strongly with diet and phylogeny (Sullam et al. 2012). Additionally, fish microbiome can modulate host immune responses to pathogenic and environmental stressors (Butt and Volkoff 2019). Thus, it is of great ecological importance to understand how the microbiome of coral reef fish assemblages responds to environmental change.

In Australia, over ~150 coral reef fish species have been observed range shifting into nearshore marine temperate ecosystems of the southeast Australian coastline during summer months (Booth et al. 2011; Feary et al. 2014). Many of these coral reef fishes interact with local temperate fishes (Smith et al. 2018; Coni, Booth, and Nagelkerken 2021), which can both increase (Smith et al. 2018; Paijmans et al. 2020) and decrease (Coni, Booth, and Nagelkerken 2021) the behavioural performance of coral reef fishes in novel temperate ecosystems. Yet, many coral reef fishes still fail to permanently establish in southeast Australian temperate ecosystems, because winter temperatures fall below their thermal critical minima and prevent overwintering success (Figueira et al. 2009; Booth et al. 2011). Additionally, these temperate ecosystems introduce novel prey, predators and competitors (Beck et al. 2016; Coni, Booth, and Nagelkerken 2021). In response, coral reef fishes can show behavioural (Coni, Booth, and Ferreira et al. 2021), dietary (Kingsbury et al. 2019; Monaco et al. 2020), habitat (Hayes et al. 2024) or physiological (Kingsbury et al. 2020; Hayes et al. 2024) plasticity to enhance their establishment in temperate ecosystems or to reduce competition with the local temperate fish species. Such responses to enhance coral reef fish persistence in their novel temperate ecosystems may be strongly shaped by their microbiome structure and plasticity, although this remains unknown.

Here we investigate the stomach microbiome of a range‐extending coral reef fish collected in situ in a global warming hotspot along a 2000‐km latitudinal gradient encapsulating their tropical core range and their temperate novel leading range edge in eastern Australia. We chose the most prevalent and successful range‐extending coral reef fish species, the sergeant major damselfish (Abudefduf vaigiensis; Hayes et al. 2024). Understanding the degree of plasticity in the stomach microbiome of tropical range‐extending fishes remains unknown but is a critical component in predicting the rate and success of their range extension and population dynamics in temperate ecosystems under future climate change.

## 2 Methods

### 2.1 Study Location and Fish Collection

**FIGURE 1:**
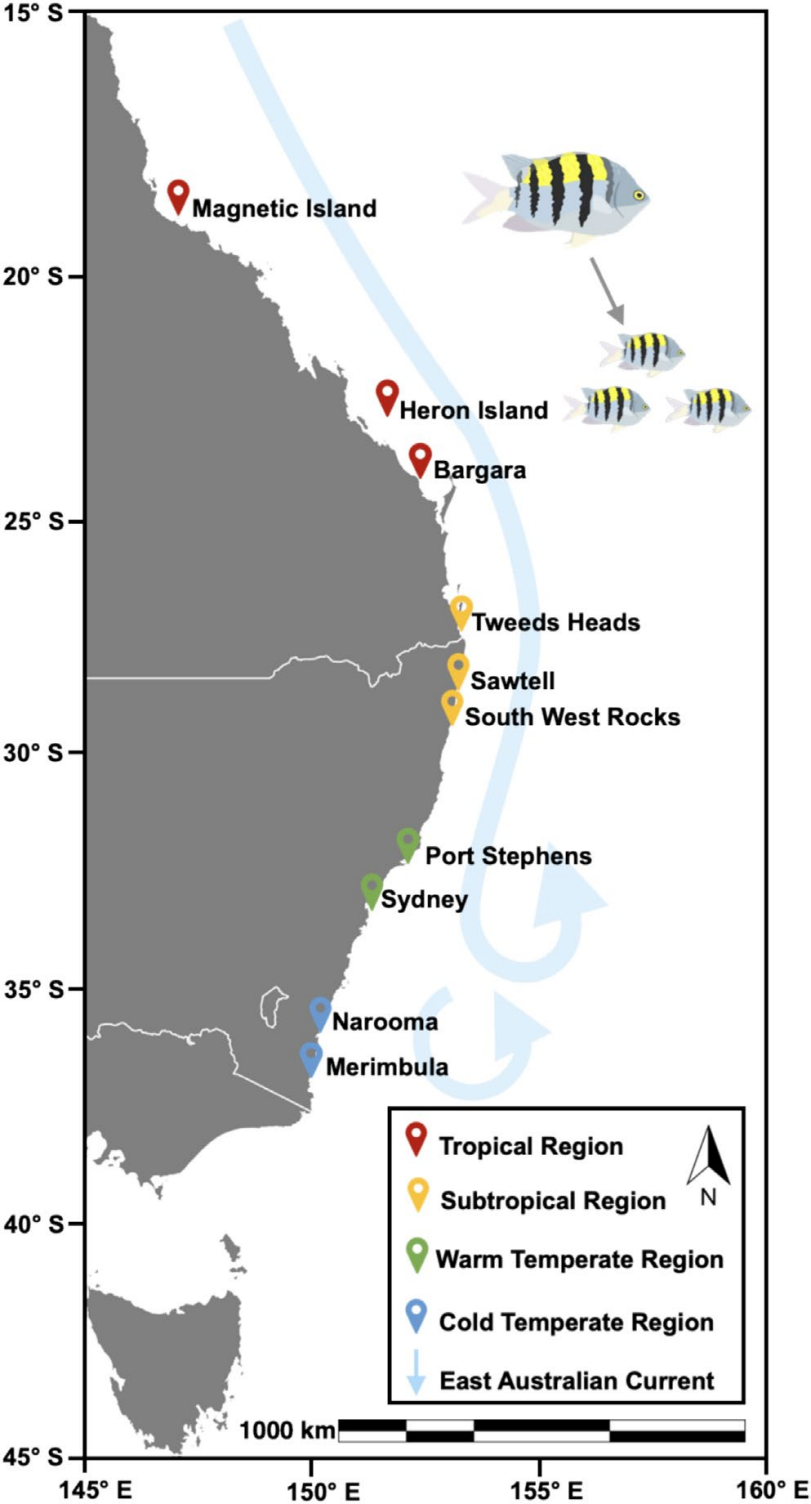
Map showing the fish collection sites and regions along the east Australian coastline encapsulating the core and leading range edges of the range‐extending coral reef fish *Abudefduf vaigiensis*. Red location points represent the core tropical region (latitude range: 19.1°–23.4° S), yellow location points represent the subtropical region (latitude range: 28.2°–30.9° S), green location points represent the warm temperate region (latitudinal range: 32.7°–33.8° S) and the blue location points represent the novel cold temperate region (latitudinal range: 36.2°–36.9° S). The blue arrow indicates the direction of the East Australian Current that annually disperses tropical coral reef fish larvae from the tropical and subtropical regions into the temperate regions.

The target fish species (*A. vaigiensis*) was collected within four range regions along a 2000‐km latitudinal gradient off the east Australian coastline during April–July 2021 to encapsulate their core and leading range edges (Figure 1). Sampling was conducted starting from high and moving to low latitudes (temperate to tropical reefs, Figure 1) to avoid seasonal biases; thus, the tropical region was sampled when water temperatures were similar to the temperate region (Table S1). The tropical region (latitudinal range: 19.1°–23.4° S) reflects the core range of this species. The subtropical region (latitudinal range: 28.2°–30.9° S) reflects a mixing zone and the southernmost point this species reproduces and overwinters (Figueira et al. 2009). The warm temperate region (latitudinal range: 32.7°–33.8° S) is considered a tropicalisation hotspot where this species has been observed range shifting for longer periods of time but fails to overwinter. The cold temperate region (latitudinal range: 36.2°–36.9° S) is the most novel and leading range edge where it also fails to overwinter (Booth et al. 2007). Juvenile fishes were collected using 9:1 ethanol:clove oil spray with handheld nets; then their associated habitat type was recorded (turf, barren, oyster, kelp and coral) because this species is site attached. Fishes were then euthanised using the *iki jime* method (Diggles 2016), and then their wet weight (±0.01 g) and standard length (±0.01 mm) were recorded (see Table S1 for replicates, mean body size and water temperature). Fish were first sprayed with 70% ethanol to avoid cross‐contamination of skin and stomach microbial communities, and whole stomachs (including the stomach contents, but excluding the oesophagus and intestines) were dissected and then stored in DNA/RNA Shield. Fish stomachs were kept frozen in DNA/RNA Shield at −20°C during field collection and then stored at −80°C until further processing. Data for physiology measures, TAC (total antioxidant capacity) and MDA (malondialdehyde concentration), were sourced from Hayes et al. (2024), which were quantified using Elabscience assay kits (catalogue numbers: E‐BC‐K168‐S, E‐BC‐K136‐S and E‐BC‐K025‐S) on the same fish as this study. TAC is an indicator of cellular defence and MDA is an indicator of cellular damage (Rodriguez‐Dominguez et al. 2019), and collectively low TAC and high MDA indicate high oxidative stress. This species shows high oxidative stress through decreased cellular defence (TAC) and increased cellular damage (MDA) towards its cold range edge (Hayes et al. 2024). Therefore, these measures were included to assess whether the observed reduction in physiological performance might be associated with shifts in microbiome structure.

### 2.2 Microbiome DNA Extraction

DNA was extracted from fish stomachs using the Maxwell RSC Faecal microbiome DNA kit following the manufacturer's instructions, with minor modifications to increase yield. In all, 500 μL of lysis buffer, 40 μL Pro‐K and 100 μL DNA/RNA Shield were added along with 15–25 mg of stomach tissue to ZR BashingBead Lysis Tubes (0.1 and 2.0 mm). Five rounds of bead beating were conducted using an MP Fast‐prep bead beater and consisted of 6.5 m/s for 1 min each at 3 min intervals on ice. Extraction blank controls were extracted alongside each extraction batch. Zymo Bacterial Community Standard was included as a positive control. DNA concentration was quantified using a Qubit Fluorometer (dsDNA High‐Sensitivity Assay Kit).

### 2.3 PCR Amplification and Sequencing

The V3 and V4 hypervariable regions of the 16S ribosomal rRNA gene were amplified using Illumina primers 341F: 5′ TCG TCG GCA GCG TCA GAT GTG TAT AAG AGA CAG CCT ACG GGN GGC WGC AG and 805R: 5′ GTC TCG TGG GCT CGG AGA TGT GTA TAA GAG ACA GGA CTA CHV GGG TAT CTA ATC C. Polymerase chain reaction (PCR) was performed on samples with each reaction consisting of 20 μL Q5 HotStart Polymerase, 0.4 μL of each primer at 20 μM, 17.6 μL nuclease‐free water and 1.6 μL of template DNA. ZymoBIOMICS Microbial Community DNA Standard was included as a positive control. Cycling conditions were as follows: (i) 98°C for 30 s; (ii) 35 cycles at 98°C for 10 s, 60°C for 30 s and 72°C for 30 s and (iii) a final elongation step at 72°C for 2 min. The DNA library was prepared following the Illumina 16S metagenomic sequencing protocol. The libraries were sequenced at the Okinawa Institute of Science and Technology (OIST, Japan) Sequencing Section on an Illumina MiSeq platform using a V3, 600 cycle kit with paired‐end reads of 300 bp length.

### 2.4 Sequence Analysis

Processing of 16S rRNA raw sequence data was performed using R statistical software (version 4.3.1; R Core Team 2023; RStudio Team 2023). Forward and reverse sequences were filtered and trimmed and potential chimeras were eliminated using DADA2 version 1.28 (Callahan et al. 2016). Sequences were excluded from analysis if they exceeded three expected errors for the forward sequence or four expected errors for the reverse sequence (maxEE = 3 and 4, respectively). Additionally, any sequences containing ambiguous nucleotides (maxN = 0) or bases with a high probability of erroneous assignment (truncQ = 2) were removed. Forward and reverse reads were trimmed to 240 bp to remove low‐quality tails. Filtered reads were dereplicated and amplicon sequence variants (ASVs) were aligned. Paired‐end reads were then merged, and chimeras were removed. Taxonomy was assigned to each ASV using the SILVA v.138.1 reference database (Quast et al. 2013; Yilmaz et al. 2014). ASVs identified as mitochondria or chloroplast, and unidentified at the phylum level, were also removed. One sample was removed from the analysis due to very low sequence reads (Sample ID: A18SY‐LM) resulting in a final sample size of 169 individuals. See Figure S1 for rarefaction curves.

### 2.5 Statistical Analysis

Statistical analysis was conducted using packages *Phyloseq* version 1.44 (McMurdie and Holmes 2013) and *Vegan* version 2.6‐4 (Oksanen et al. 2007), and graphical outputs were used with package *ggplot2* version 3.4.2 (Wickham 2016). For alpha diversity analysis, we calculated ASV richness (number of bacterial species observed) and community evenness (distributional equity of ASV abundances, calculated as Shannon diversity divided by log richness) for each sample using the *estimate_richness* function in the package *Phyloseq*. Levene's test indicated that both alpha diversity measures were not homogeneous and normally distributed; therefore, non‐parametric Kruskal–Wallis and Dunn tests were performed on log‐transformed data to determine significance between regions. Due to no differences across sites within the regions and low sample size within sites, analyses were performed at the region level as a fixed factor. To account for potential body‐size effects of naturally smaller fish at higher latitudes, water temperature and physiological effects, we ran linear regressions to quantify both alpha diversity measures trends in response to wet weight of the fish, water temperature and physiology (because this species experiences physiological stress at high latitudes; Hayes et al. 2024). Kruskal–Wallis and Dunn tests were then performed to determine if the alpha diversity measures were influenced by habitat preference (because *A. vaigiensis* is a site‐attached species). Beta diversity community measures were calculated using the *distance* function in the *Phyloseq* package and were tested with a range of phylogenetic and non‐phylogenetic dissimilarity measures that weigh the relative abundance of ASVs differently to recognise the effect of abundant ASVs (non‐phylogenetic: Bray–Curtis, and phylogenetic: weighted UniFrac and unweighted UniFrac). Bray–Curtis dissimilarity measures differences in the relative abundance of ASVs without incorporating their phylogenetic relationships, whereas weighted and unweighted UniFrac metrics account for phylogenetic relationships between ASVs. Weighted UniFrac considers both the relative abundance and phylogenetic distances of ASVs, making it sensitive to differences in dominant taxa, and unweighted UniFrac considers the presence or absence of ASVs, which highlights differences in rare taxa regardless of their abundance. To determine differences in beta diversity measures across regions, permutational multivariate analysis of variance (PERMANOVA, using functions *adonis2* and *pairwise.adonis* in the package *Vegan*) was computed with 9999 permutations and square‐root–transformed data. Microbiome variability was calculated using permutational analysis of multivariate dispersion (PERMDISP2, using function *betadisper* in the package *Vegan*) on the transformed data with bias correction to account for differences in sample sizes among regions. Dispersion measures the homogeneity of variance between groups and compares the average distance to the centroid within each group in multidimensional space. Principal coordinate analysis (PCoA) was used to visualise dissimilarities between regions and determine whether differences are influenced by multivariate dispersion or the regions. Stacked bar graphs were plotted at the genus level and represent the top 10 most abundant taxa across the sampling regions. Relative abundances were calculated within each sample and then averaged across samples for each region. To detect ASVs that were differently abundant (significantly different), we used the analysis of compositions of microbiomes with bias correction 2 (ANCOMBC‐2; Lin and Peddada 2020; Lin et al. 2022) and reported the effect with log fold change (LFC), the magnitude of differential abundance across sampling regions at the genus level. We considered genera to be differently abundant when the false discovery rate (FDR)–corrected *p*‐value (*q*‐value) was less than 0.05.

## 3 Results

### 3.1 Sequencing Data Summary and Depth

Sequencing of the 16S rRNA gene V3–V4 region of 169 samples resulted in 11,172,401 raw sequences obtained in the final dataset. After quality filtering, denoising and merging of paired‐end reads, a total of 7,894,700 non‐chimeric reads for the samples were obtained and ranged from 17,499 to 170,063 reads per sample. The total number of ASVs detected for the samples was 21,415.

### 3.2 Alpha Diversity and Evenness of the Stomach Microbiome Across Regions

**FIGURE 2:**
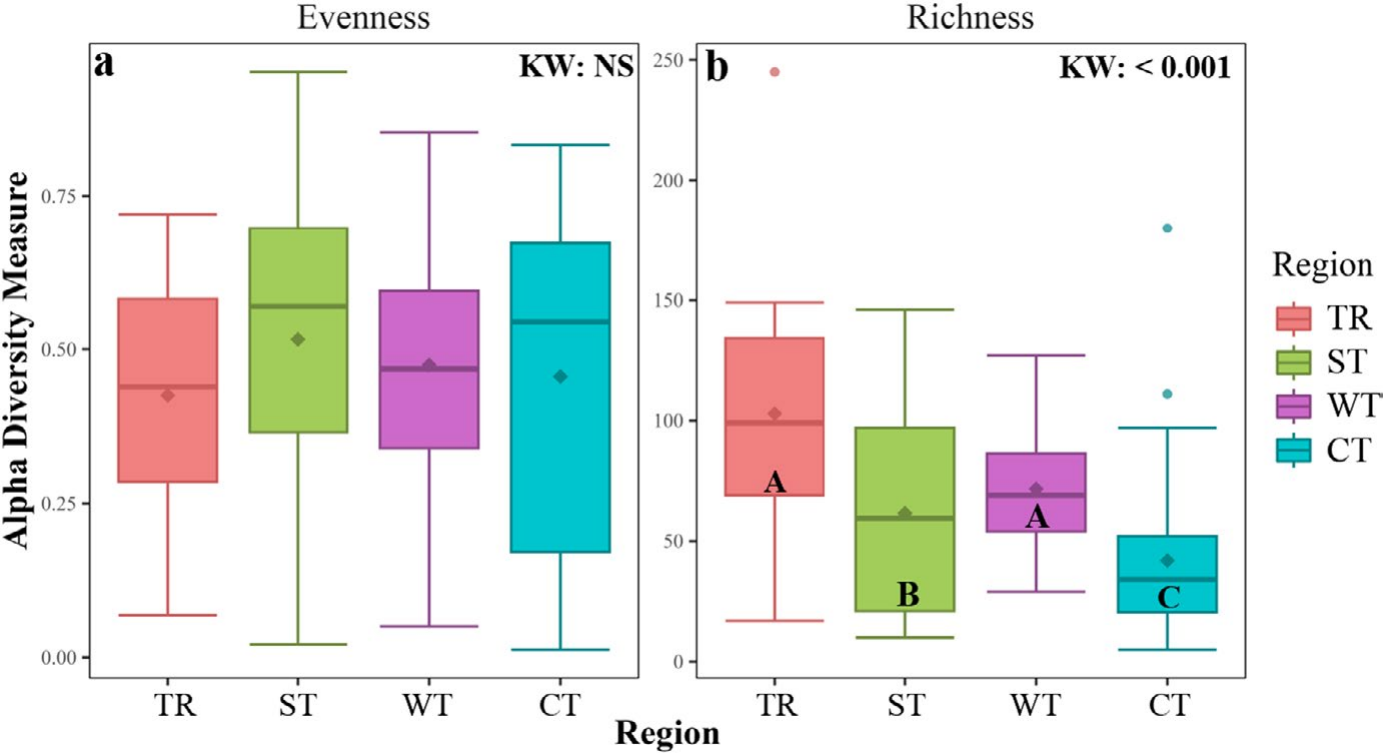
Boxplots showing differences in alpha diversity measures evenness (a) and richness (b) of *Abudefduf vaigiensis* microbiome across the different climatic regions (TR, tropical; ST, subtropical; WT, warm temperate; CT, cold temperate). The boxes represent the lower and upper quartiles, whiskers represent the extremum values, horizontal lines show the median and diamonds show the mean. KW indicates the degree of significance for the main test (Kruskal–Wallis test) and the different letters indicate the Dunn's *post hoc* test's significant differences between regions (*p* < 0.05).

Stomach microbial community evenness showed no differences across climatic regions (Figure 2a, *p* = 0.509, Table S2), but microbiome richness significantly differed across regions (Figure 2b, *p* < 0.001, Table S2). Richness was lower in fish collected at their novel cold temperate range compared to their warm temperate (Figure 1b; *p* < 0.001, Table S2), subtropical (*p* = 0.008; Table S2) and tropical (*p* < 0.001, Table S2) ranges, but did not differ between tropical and warm temperate regions (*p* = 0.117, Table S2).

### 3.3 Beta Diversity of the Stomach Microbiome Across Regions

**FIGURE 3:**
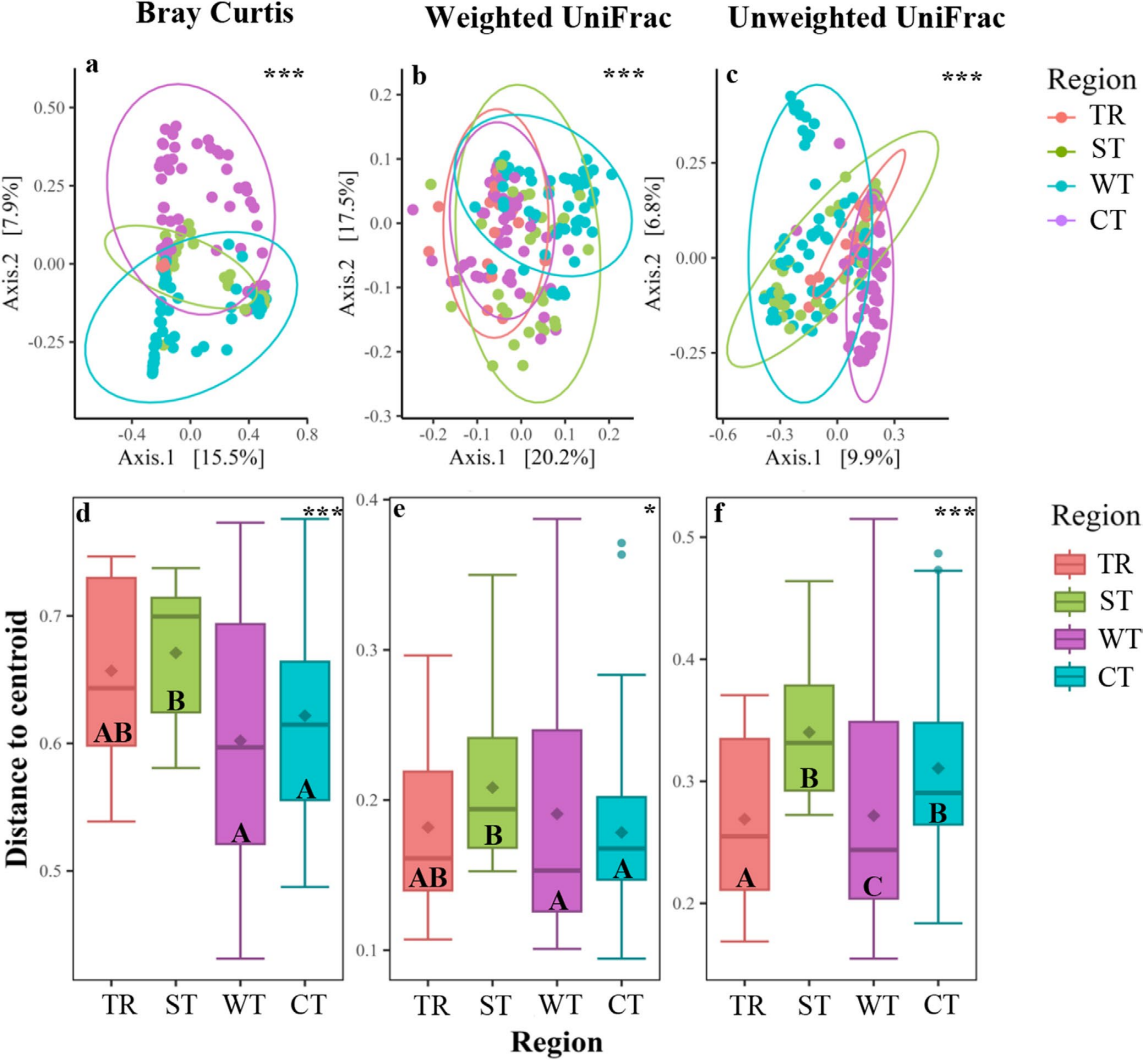
Principal coordinate analysis (PCoA) showing microbiome composition (a–c) and compositional variability (distance to centroid; d–f) for range‐extending coral reef fish species *Abudefduf vaigiensis* across the different sampling regions (TR, tropical; ST, subtropical; WT, warm temperate; CT, cold temperate). Significance in panels (a–c) depicts differences in permutational analysis of variance (PERMANOVA). Compositional variability represents the distance to the centroid in multivariate space, and the boxes represent the lower and upper quartiles, whiskers represent the extremum values, horizontal lines show the median and diamonds show the mean. Different letters in panels (d–f) indicate differences in multivariate dispersion between regions. The coefficient * indicates that the *p*‐value is < 0.05 and *** that the *p*‐value is < 0.001. Multivariate analyses were computed with non‐phylogenetic (Bray–Curtis: a, d) and phylogenetic measures (weighted Unifrac: b, e; unweighted Unifrac: c, f). Bray–Curtis considers ASV abundance data, weighted Unifrac considers ASV abundance data in consideration of phylogenetic positioning and unweighted Unifrac considers presence–absence of data in consideration to phylogenetic positioning.

Beta diversity of the microbiota differed across climate regions for Bray–Curtis (Figure 3a, *p* < 0.001, Table S3), weighted UniFrac (Figure 3b, *p* < 0.001, Table S3) and unweighted UniFrac (Figure 3c, *p* < 0.001, Table S3) metrics. Multivariate dispersion (variability) of microbiome composition differed among climate regions but without revealing a consistent pattern, for Bray–Curtis (Figure 3d, *p* < 0.001, Table S4), weighted UniFrac (Figure 3e, *p* = 0.040, Table S4) and unweighted UniFrac (Figure 3f, *p* < 0.001, Table S4) metrics. However, for Bray–Curtis, warm temperate and cold temperate regions showed lower variability compared to the subtropical region, but not compared to the tropical region (pairwise: *p* < 0.001, Table S4). For the weighted UniFrac, warm temperate and cold temperate regions showed lower variability than the subtropical region (pairwise; *p* < 0.004, Table S4), while for unweighted UniFrac, subtropical, warm temperate and cold temperate regions showed higher microbial variability compared to the tropical region (pairwise; *p* < 0.004, Table S4).

### 3.4 Taxonomic Composition of the Stomach Microbiome

**FIGURE 4:**
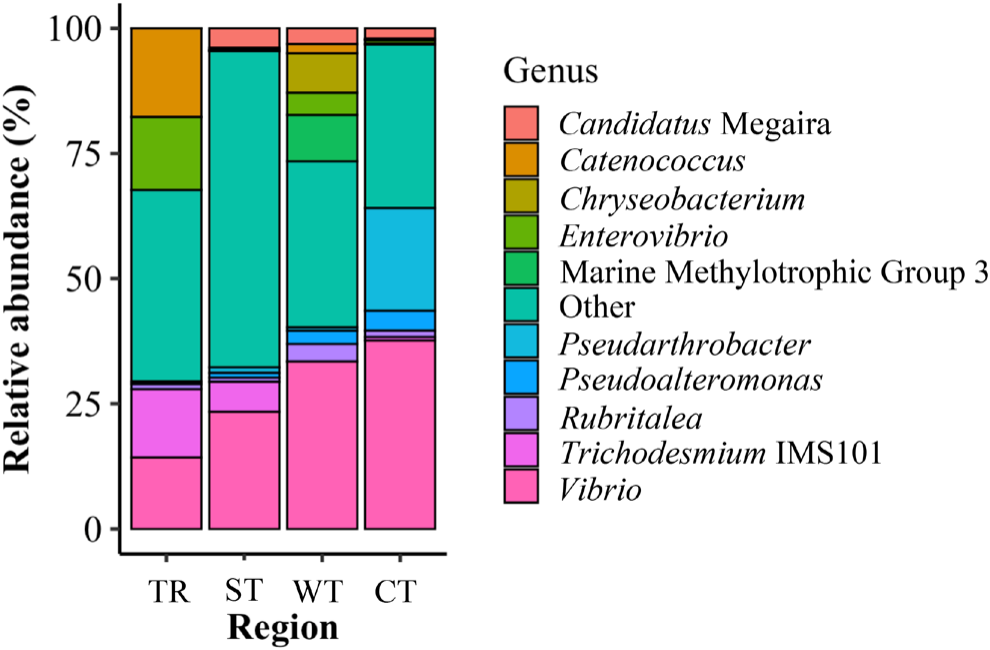
Stacked bar plot showing relative abundance (%) of bacterial genera in the microbiome of a range‐extending coral reef fish species *Abudefduf vaigiensis* across the sampling regions (TR, tropical; ST, subtropical; WT, warm temperate; CT, cold temperate).

**FIGURE 5:**
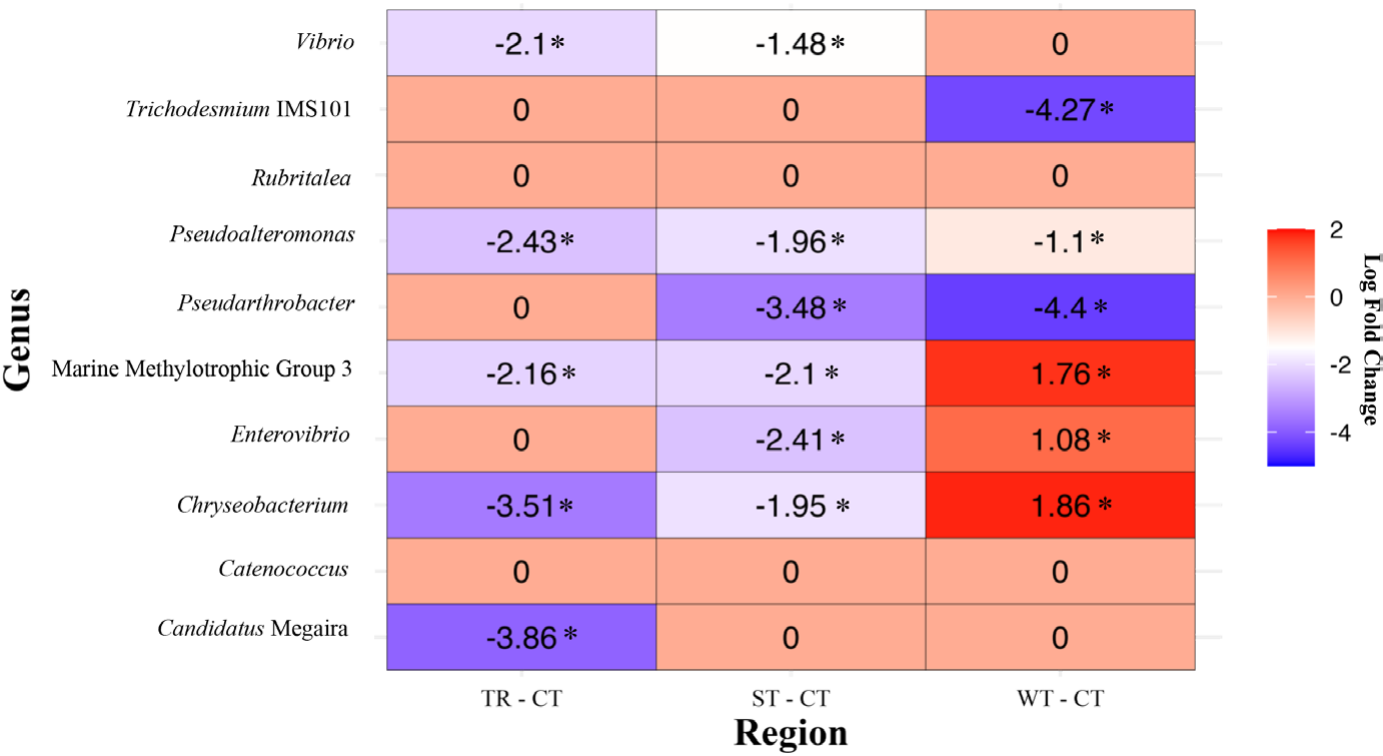
Heat map showing the magnitude (log fold change) of significantly different microbiome genera of a range‐extending coral reef fish species *Abudefduf vaigiensis* across sampling regions (TR, tropical; ST, subtropical; WT, warm temperate; CT, cold temperate). The regions were compared against the cold temperate region as the reference variable. Significant differences in abundance of the genera are shown by * (FDR *q* < 0.05). The red cells show increased abundance (log fold change > 0) and the blue cells show decreased abundance (log fold change < 0) compared to the cold temperate range.

The microbiome of fishes collected at the cold temperate region was dominated by the genera *Vibrio* (relative abundance ± SE: 37.7% ± 5.2%) and *Pseudarthrobacter* (20.5% ± 3%). At the warm temperate region, the microbiome was dominated by *Vibrio* (33.4% ± 4.4%) and Marine Methylotrophic Group 3 (9.2% ± 2%). At the subtropical region, the microbiome was dominated by *Vibrio* (23.4% ± 5.4%) and *Candidatus* Megaira (3.9% ± 1.7%). The microbiome at the tropical region was dominated by the genera *Vibrio* (14.3% ± 6.5%), *Catenococcus* (17.7% ± 6.6%), *Enterovibrio* (14.6% ± 6.7%) and *Trichodesmium* IMS101 (13.7% ± 6.3%) (Figure 4). Towards the cold temperate region, *Vibrio* and *Pseudarthrobacter* increased in relative abundance, while *Trichodesmium* IMS101 decreased compared to the tropical region (Figure 4). Eight of the top 10 genera were significantly different (FDR *q* < 0.05) between the tropical, subtropical and warm temperate regions compared to the cold temperate region. Five genera in the tropical (*Candidatus* Megaira, *Chryseobacterium*, Marine Methylotrophic Group 3, *Pseudoalteromonas* and *Vibrio*), six genera in the subtropical (*Chryseobacterium*, *Enterovibrio*, Marine Methylotrophic Group 3, *Pseudarthrobacter*, *Pseudoalteromonas* and *Vibrio*) and three genera in the warm temperate (*Pseudarthrobacter*, *Pseudoalteromonas* and *Trichodesmium* IMS101) were found in significantly lower abundances than in the cold temperate region (Figures 4 and 5; FDR *q* < 0.04; Table S5). However, three genera (*Chryseobacterium*, *Enterovibrio* and Marine Methylotrophic Group 3) showed significantly higher abundance in the warm temperate region than in the cold temperate region. Two genera (*Rubritalea* and *Catenococcus*) did not significantly differ in abundance between the cold temperate and the other regions (Figures 4 and 5; FDR *q* > 0.05, Table S5). Towards the warm temperate region, *Trichodesmium* IMS101 and *Pseudarthrobacter* decreased, whereas Marine Methylotrophic Group 3, *Chryseobacterium* and *Candidatus* Megaira increased in relative abundance compared to either the tropical or subtropical region (Figure S2, FDR *q* < 0.05, Table S5). *Pseudarthrobacter* and *Candidatus* Megaira decreased in relative abundance from the tropical region towards the subtropical region (Figure S2, FDR *q* < 0.05, Table S5).

### 3.5 Biological Factors Associated With Microbiome Diversity and Evenness

Stomach microbiome diversity (richness and evenness) was not associated with fish body size (wet weight, *p* > 0.166, *R* ^2^ < 0.14, Figure S3, Table S6) across regions, except within the subtropical and warm temperate regions, where richness and evenness increased with body size, respectively (subtropical: *p* < 0.001, *R* ^2^ = 0.33, warm temperate: *p* = 0.003, *R* ^2^ = 0.16). Cellular defence (TAC) and cellular damage (MDA) were not associated with microbiome richness or evenness across regions (*p* > 0.067, *R* ^2^ < 0.21, Figure S4, Tables S7 and S8), except for the tropical and warm temperate regions where cellular defence decreased with richness (tropical: *p* = 0.012, *R* ^2^ < 0.01, warm temperate: *p* = 0.006, *R* ^2^ = 0.14). Microbiome richness and evenness did not differ across habitat types; however, in the subtropical region, evenness was lower in oyster habitats compared to turf habitats (Dunn's test: *p* < 0.006, Figure S5c, Table S9), while in the warm temperate region, evenness and richness were lower in oyster habitats than turf and barren habitats (Dunn's test: *p* < 0.009, Figure S5e,f, Table S9). Microbiome richness and evenness were not correlated with water temperature (richness: *p* = 0.866, *R* ^2^ = 0.02, evenness: *p* = 0.517, *R* ^2^ = 0.23, Figure S6, Table S10).

## 4 Discussion

We here show that the microbiome of a prevalent range‐extending coral reef fish is simplified at its novel temperate cold‐range edge. Decreased microbiome diversity and a shift in microbiome community structure associated with an increased prevalence of opportunistic bacteria (*Vibrio*) at the cold temperate region compared to its historical range together indicate simplification and dysbiosis, respectively, of the fish stomach microbiome in their novel ranges. Although high variability in microbial community structure can also indicate dysbiosis (Zaneveld et al. 2017), we found no consistent trends of variability across sampling regions. Dysbiosis of the microbiome and increased prevalence of *Vibrio* species can negatively affect the health and fitness of range‐extending coral reef fishes by increasing immune suppression (Moore et al. 2024), disease occurrence (Belden and Harris 2007) and mortality (Greenspan et al. 2020; Risely et al. 2023). Additionally, this can compromise their behavioural (Florkowski and Yorzinski 2023) and physiological (Gould et al. 2018) responses, both of which can underpin successful range extensions into temperate ecosystems. Coral reef fishes extending their ranges into temperate ecosystems experience increased susceptibility to cold stress (Figueira et al. 2009) and novel interactions with temperate competitors, prey or predators (Beck et al. 2016); therefore, simplification and dysbiosis of their microbiome could exacerbate vulnerability to novel stressors at their leading range edge. This suggests that microbiome simplification may mediate the colonisation and persistence of range‐extending coral reef fish in novel temperate ecosystems.

Microbiome plasticity of range‐extending species may enhance their adaptive potential and persistence in novel ecosystems. At the tropical and subtropical regions, the microbiome of the range‐extending fish species showed a heterogeneous community structure, with no distinct genera dominating their stomach microbiome. However, at the novel cold temperate region, the community structure was simplified (i.e., less diverse) with two genera (*Vibrio* and *Pseudarthrobacter*) contributing to ~58% of the relative microbial abundance. Although the function of *Pseudarthrobacter* in fishes remains unknown, it is well understood that some *Vibrio* species are pathogenic and can cause body malformation, slow growth and increased disease prevalence and mortality in fishes (Ina‐Salwany et al. 2018). This change in microbiome community structure and diversity suggests the range‐extending coral reef fish exhibits low microbial plasticity at their cold‐range edge. Whilst this species shows high dietary and behavioural plasticity at their cold‐range edge (Kingsbury et al. 2019; Coni, Booth, and Ferreira et al. 2021), the observed low microbiome plasticity could reduce their ability to respond to novel challenges (competition, predation, prey and cold stress) in temperate ecosystems. Therefore, the inability to maintain the integrity of their stomach microbiome may mitigate their adaptive potential and persistence at their cold‐range edge.

Stomach microbiome diversity of the range‐extending coral reef fish was unaffected by water temperature, habitat types, cellular defence, cellular stress and body size towards their cold‐range edge compared to their tropical native range. This suggests that the observed reduced microbiome diversity at their cold‐range edge occurs independently and is not influenced by novel temperate water temperatures, habitat types or host physiological performance. Host habitat (Kim et al. 2021; Clever et al. 2022), physiology (Clements et al. 2014), diet (Miyake et al. 2014) and behaviour (Trevelline and Kohl 2022) have previously been identified as major determinants of microbial diversity. Our focal species experiences increased oxidative stress (the combination of decreased cellular defence and increased cellular damage) at their cold‐range edge compared to their tropical native range (Hayes et al. 2024), as well as reduced feeding and activity levels (Kingsbury et al. 2020; Coni et al. 2022). The increased cellular damage may diverge energy away from other important fitness‐related traits such as reproduction and growth (Birnie‐Gauvin et al. 2017; Zhang et al. 2023). This species also consumes a wide variety of prey groups across the regions and shows a high degree of dietary generalism in novel temperate ecosystems (Kingsbury et al. 2019; Monaco et al. 2020). At the cold temperate region compared to the subtropical region, consumption increased for zooplankton (from ~38% to ~66%) and crustaceans (from ~4% to ~16%), but that of macroalgae decreased (from ~50% to ~7%; see figure S7 in Kingsbury et al. 2019). The observed simplification of the microbiome community structure of the fish could be influenced by an indirect response to other environmental changes along the gradient, such as altered prey communities. Factors influencing microbiome shifts, such as water temperatures, food sources and habitat types, are expected to change and simplify under future climate change scenarios (Nagekerken et al. 2020; Agostini et al. 2020; Coni, Nagelkerken, and Ferreira et al. 2021). Direct and indirect changes in abiotic and biotic variables are both driven by changes in climate, such as rapid ocean warming at our warm and cold temperate study sites. Therefore, irrespective of the underlying mechanisms influencing the microbiome structure, the observed shifts in the microbiome of a common range‐extending coral reef fish may mediate their persistence in novel temperate ecosystems.

Future ocean warming will likely relax the thermal stress of coral reef fishes residing in novel temperate ecosystems and increase their likelihood of successful persistence, which may reduce microbial disturbances. Climate‐driven warming and strengthening of the East Australian Current (Wu et al. 2012) are projected to expand the prevalence of tropical microbes into temperate waters (Messer et al. 2020), potentially mediating beneficial microbial taxonomic shifts that could relieve current dysbiosis in their microbial structure. However, ocean warming drives higher abundances of *Vibrio* species (Baker‐Austin et al. 2013) because their abundance is positively correlated with increasing water temperature (Williams et al. 2022). Despite this, tropicalisation of microbial communities in temperate ecosystems could introduce beneficial microbes capable of suppressing pathogenic *Vibrio* (Messer et al. 2020), although this remains unknown. Additionally, ocean warming can benefit range‐extending coral reef fishes in temperate ecosystems through increased physiological function (Mitchell et al. 2023a), growth (Djurichkovic et al. 2019; Mitchell et al. 2023b) and foraging performance (Coni, Booth, and Nagelkerken 2021), overall enhancing successful establishment in their future ecosystems. Therefore, when future water temperatures track the thermal optima of range‐extending coral reef fishes, negative alterations to microbial communities may be alleviated and benefit the establishment of coral reef fishes in temperate ecosystems.

## 5 Conclusions

We reveal that the stomach microbiome of a prevalent range‐extending coral reef fish shows decreased diversity and increased abundance of pathogenic bacterial species, which indicates dysbiosis and low plasticity of their microbiome at their novel temperate cold‐range edge. Dysbiosis and low plasticity of the microbiome might be a present‐day mediator of the rate of colonisation and persistence of coral reef fishes in the early stages of range extensions into temperate ecosystems, irrespective of the immediate drivers of gastrointestinal microbiome changes.

## Author Contributions

C.H., A.M., and I.N. conceived and designed the study. C.H., A.M., I.N. and D.J.B. conducted the fieldwork. J.J. and C.L. extracted the DNA and prepared it for sequencing. T.R. contributed reagents and supplies. C.H. and R.H. analysed the data. C.H. wrote the manuscript with the help from all authors. All authors reviewed the manuscript and contributed to the final version.

## Ethics Statement

This experiment was conducted according to the University of Adelaide Animal Ethics and University of Technology Sydney guidelines and permits S‐2020‐13 and 2017–1117, respectively, and under New South Wales DPI Scientific Collection Permit F94/696(A)‐9.0 and Great Barrier Reef Marine Park Permits G20/43958.1 and G21/45557.1.

## Conflicts of Interest

The authors declare no conflicts of interest.

## Supporting information


Data S1.

## Data Availability

The data that support the findings of this study are openly available in figshare: http://doi.org/10.25909/24780432. Raw sequence reads are uploaded and available on the NCBI Sequence Read Archive (PRJNA1089790).
