# Supplementary material for: Stomach Microbiome Simplification of a Coral Reef Fish at Its Novel Cold‐Range Edge Under Climate Change
Source: Mol Ecol. 2025 Feb 22;34(7):e17704. doi: 10.1111/mec.17704 (PMC11934084; doi:10.1111/mec.17704)
Supplement: Supplementary file 1 — Data S1. [file MEC-34-e17704-s001.docx]

**Supplementary material**

**Table S1:** Replicates and mean ± standard error (SE) for wet weight (WW in g) and standard length (SL in mm) measurements used for stomach microbiome analysis of range-extending coral reef fish species *A. vaigiensis.* Average seawater temperatures (°C) were collected using a dive watch at the time of fish collection.

| **Region** | **Sample size** | **WW (mean ± SE g)** | **SL (mean ± SE mm)** | **Temperature (°C)** |
| --- | --- | --- | --- | --- |
| Tropical | 16 | 1.20 ± 0.16 | 25.92 ± 0.90 | 21.6 |
| Subtropical | 40 | 1.18 ± 0.16 | 25.80 ± 0.88 | 22.8 |
| Warm temperate | 55 | 1.27 ± 0.18 | 26.46 ± 0.96 | 20.6 |
| Cold temperate | 59 | 1.21 ± 0.16 | 25.97 ± 0.91 | 21.6 |
| Total | 170 |  |  |  |


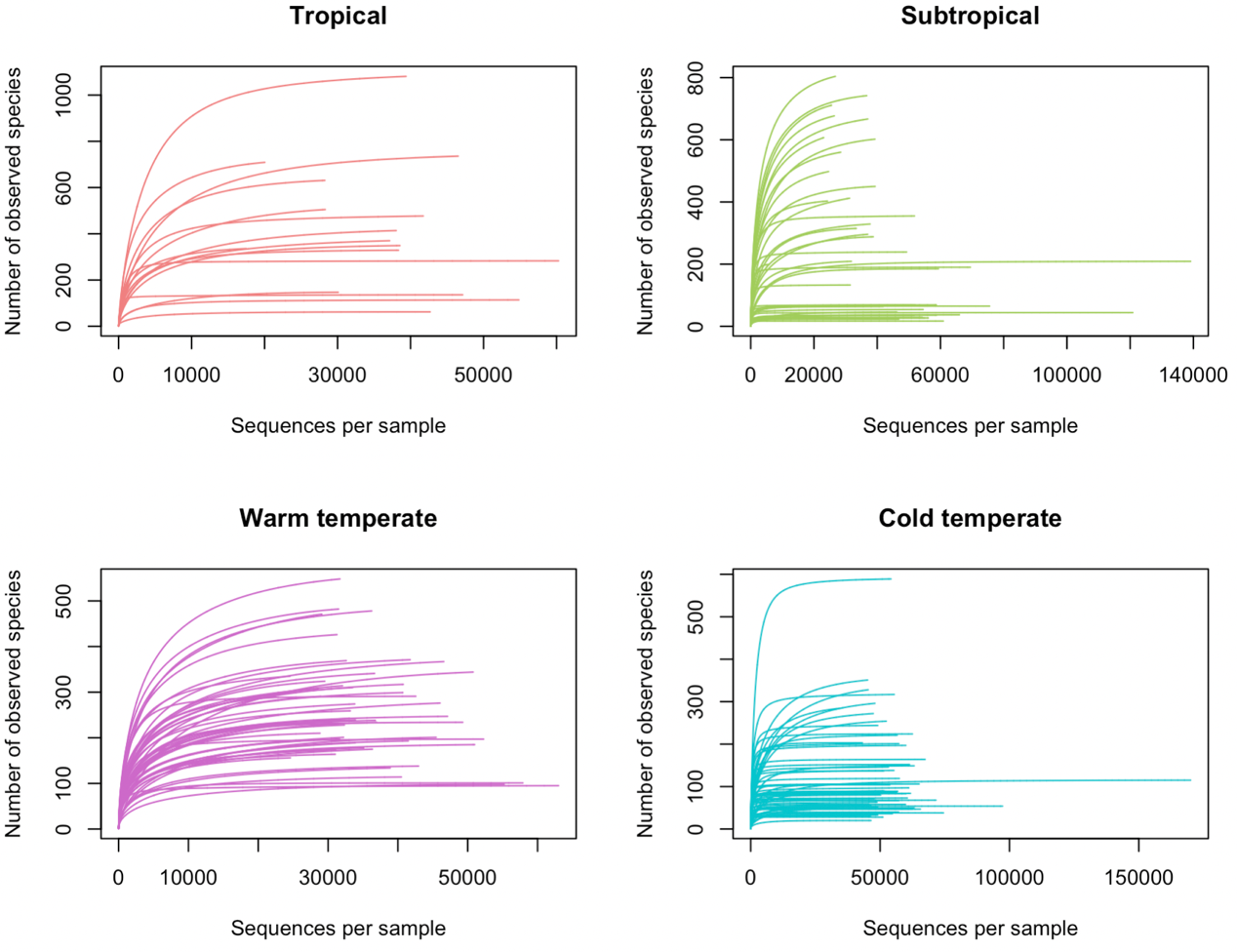


**Figure S1:** Rarefaction curves for total number of bacterial species observed and number of sequences per sample.

**Table S2:** Kruskal-Wallis and Dunn-tests for alpha diversity measures richness and evenness across sampling regions: tropical (TR), subtropical (ST), warm-temperate (WT) and cold-temperate (CT). Significant p-values shown in bold (< 0.05). Tests were computed with 9,999 permutations.

| **Kruskal-Wallis** | | | | | | |
| --- | --- | --- | --- | --- | --- | --- |
| Diversity | | Kruskal-Wallis χ^2^ | | df | p-value | |
| Richness | | 38.311 | | 3 | **< 0.001** | |
| Evenness | | 2.320 | | 3 | 0.509 | |
| **Post-hoc Dunn-test** | | | | | | |
| Diversity | Region | | Z | | p-value |  |
| Richness  Richness  Richness  Richness  Richness  Richness | TR - ST | | -2.90 | | **0.004** |  |
|  | TR - WT | | -1.57 | | 0.117 |  |
|  | TR - CT | | -4.98 | | **< 0.001** |  |
|  | ST - WT | | 1.97 | | **0.049** |  |
|  | ST - CT | | -2.66 | | **0.008** |  |
|  | WT - CT | | -5.07 | | **< 0.001** |  |

**Table S3:** PERMANOVA and pairwise tests for beta diversity measures Bray Curtis, weighted UniFrac and unweighted UniFrac across the sampling regions: tropical (TR), subtropical (ST), warm temperate (WT) and cold temperate (CT). Significant values (< 0.05) shown in bold. Tests were computed with 9,999 permutations.

| **Bray Curtis** | | | | | |
| --- | --- | --- | --- | --- | --- |
| **PERMANOVA** | | | | | |
|  | Df | Sum Sq | R^2^ | f-value | p-value |
| Groups | 3 | 7.460 | 0.101 | 6.146 | **< 0.001** |
| Residual | 165 | 66.759 | 0.899 |  |  |
| Total | 168 | 74.219 | 1.000 |  |  |
| **Pairwise PERMANOVA** | | | | | |
| Region | Df | Sum Sq | F model | R^2^ | p-value |
| ST-TR | 1 | 1.421 | 3.169 | 0.055 | **0.001** |
| ST-CT | 1 | 1.881 | 4.512 | 0.044 | **0.001** |
| ST-WT | 1 | 2.368 | 5.815 | 0.059 | **0.001** |
| TR-CT | 1 | 2.316 | 5.771 | 0.073 | **0.001** |
| TR-WT | 1 | 2.458 | 6.347 | 0.085 | **0.001** |
| CT-WT | 1 | 3.776 | 9.851 | 0.082 | **0.001** |
| **Weighed UniFrac** | | | | | |
| **PERMANOVA** | | | | | |
|  | Df | Sum Sq | R^2^ | f-value | p-value |
| Groups | 3 | 1.339 | 0.132 | 8.335 | **< 0.001** |
| Residual | 165 | 8.836 | 0.868 |  |  |
| Total | 168 | 10.175 | 1.000 |  |  |
| **Pairwise PERMANOVA** | | | | | |
| Region | Df | Sum Sq | F model | R^2^ | p-value |
| ST-TR | 1 | 0.199 | 3.260 | 0.057 | **0.004** |
| ST-CT | 1 | 0.409 | 6.930 | 0.067 | **0.001** |
| ST-WT | 1 | 0.388 | 7.012 | 0.071 | **0.001** |
| TR-CT | 1 | 0.463 | 9.003 | 0.110 | **0.001** |
| TR-WT | 1 | 0.236 | 5.175 | 0.071 | **0.001** |
| CT-WT | 1 | 0.770 | 15.404 | 0.122 | **0.001** |
| **Unweighed UniFrac** | | | | | |
| **PERMANOVA** | | | | | |
|  | Df | Sum Sq | R^2^ | f-value | p-value |
| Groups | 3 | 5.858 | 0.093 | 5.645 | **< 0.001** |
| Residual | 165 | 56.070 | 0.907 |  |  |
| Total | 168 | 62.928 | 1.000 |  |  |
| **Pairwise PERMANOVA** | | | | | |
| Region | Df | Sum Sq | F model | R^2^ | p-value |
| ST-TR | 1 | 0.982 | 2.684 | 0.047 | **0.001** |
| ST-CT | 1 | 1.255 | 3.292 | 0.033 | **0.001** |
| ST-WT | 1 | 2.191 | 6.808 | 0.069 | **0.001** |
| TR-CT | 1 | 1.574 | 4.186 | 0.054 | **0.001** |
| TR-WT | 1 | 1.476 | 5.000 | 0.068 | **0.001** |
| CT-WT | 1 | 3.391 | 10.093 | 0.083 | **0.001** |

**Table S4:** PERMANOVA and pairwise outputs for beta dispersion measures on Bray Curtis, weighted UniFrac and unweighted UniFrac distances. Significant values (<0.05) indicate samples unevenly dispersed (i.e., high variability).

| **Bray Curtis** | | | | | |
| --- | --- | --- | --- | --- | --- |
| **Beta dispersion: PERMANOVA** | | | | | |
|  | Df | Sum Sq | Mean Sq | f-value | p-value |
| Groups | 3 | 0.124 | 0.041 | 6.173 | **< 0.001** |
| Residuals | 165 | 1.107 | 0.007 |  |  |
| **Beta dispersion: Pairwise** | | | | | |
|  | CT | ST | T | WT |  |
| CT | - | **< 0.001** | 0.109 | 0.265 |  |
| ST | **< 0.001** | - | 0.420 | **< 0.001** |  |
| TR | 0.104 | 0.422 | - | 0.054 |  |
| WT | 0.258 | **< 0.001** | 0.054 | - |  |
| **Weighted UniFrac** | | | | | |
| **Beta dispersion: ANOVA** | | | | | |
|  | Df | Sum Sq | Mean Sq | f-value | p-value |
| Groups | 3 | 0.048 | 0.016 | 2.735 | **0.040*** |
| Residuals | 165 | 0.961 | 0.006 |  |  |
| **Beta dispersion: Pairwise** | | | | | |
|  | CT | ST | T | WT |  |
| CT | - | **0.004** | 0.803 | 0.885 |  |
| ST | **0.006** | - | 0.080 | **0.013** |  |
| TR | 0.800 | 0.080 | - | 0.772 |  |
| WT | 0.888 | **0.014** | 0.767 | - |  |
| **Unweighted UniFrac** | | | | | |
| **Beta dispersion: ANOVA** | | | | | |
|  | Df | Sum Sq | Mean Sq | f-value | p-value |
| Groups | 3 | 0.279 | 0.093 | 45.107 | **< 0.001** |
| Residuals | 165 | 0.340 | 0.002 |  |  |
| **Beta dispersion: Pairwise** | | | | | |
|  | CT | ST | T | WT |  |
| CT | - | 0.257 | **< 0.001** | **< 0.001** |  |
| ST | 0.254 | - | **0.004** | **< 0.001** |  |
| TR | **< 0.001** | **0.003** | - | **0.002** |  |
| WT | **< 0.001** | **< 0.001** | **0.002** | - |  |


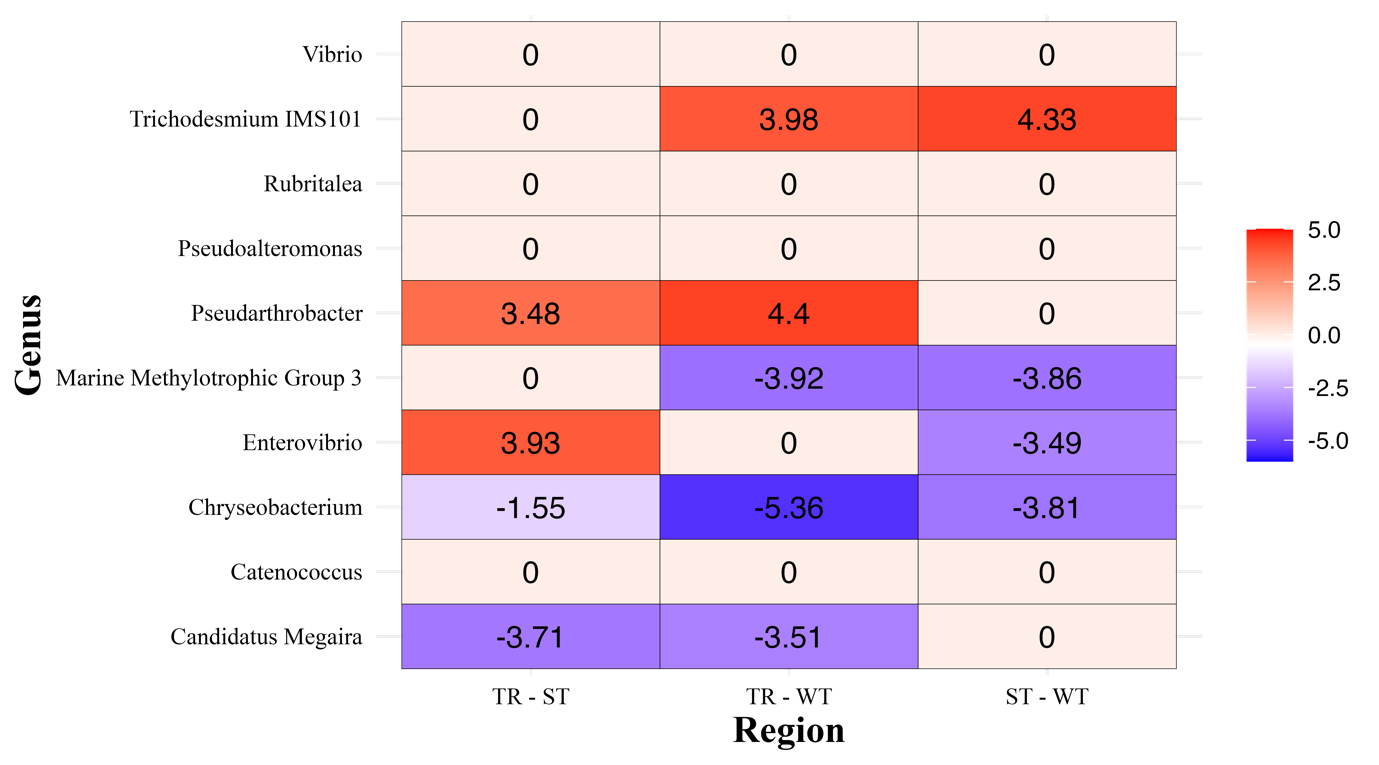


*

*

*

*

*

*

*

*

*

*

*

*

*Vibrio*

*Trichodesmium* IMS101

*Rubritalea*

*Pseudoalteromonas*

*Pseudarthrobacter*

Marine Methylotrophic Group 3

*Enterovibrio*

*Chryseobacterium*

*Catenococcus*

*Candidatus* Megaira

**Log Fold Change**

*

Figure S2: Heatmap showing the magnitude (log fold change) of significantly different stomach microbiome genera of a range-extending coral reef fish across sampling regions (TR = tropical, ST = subtropical, WT = warm temperate, CT = cold temperate). Significant differences in abundance of the genera are shown by * (FDR q < 0.05). The red cells show increased abundance (log fold change > 0), and the blue cells show decreased abundance (log fold change < 0).

| **Taxon** | **Log fold change** | | | | | | **Standard error** | | | | | |
| --- | --- | --- | --- | --- | --- | --- | --- | --- | --- | --- | --- | --- |
|  | TR-CT | ST-CT | WT-CT | TR-ST | TR-WT | ST-WT | TR-CT | ST-CT | WT-CT | TR-ST | TR-WT | ST-WT |
| *Vibrio* | -2.098 | -1.480 | -1.232 | -0.617 | -0.866 | -0.248 | 0.685 | 0.519 | 0.493 | 0.751 | 0.734 | 0.588 |
| *Pseudarthrobacter* | 0.000 | -3.484 | -4.404 | 3.484 | 4.404 | 0.920 | 0.476 | 0.394 | 0.362 | 0.495 | 0.477 | 0.419 |
| Marine Methylotrophic Group 3 | -2.160 | -2.104 | 1.758 | -0.056 | -3.917 | -3.862 | 0.487 | 0.322 | 0.392 | 0.463 | 0.525 | 0.423 |
| *Trichodesmium* IMS101 | -0.289 | 0.062 | -4.273 | -0.350 | 3.984 | 4.334 | 0.786 | 0.409 | 0.298 | 0.790 | 0.749 | 0.403 |
| Candidatus Megaira | -3.855 | -0.142 | -0.344 | -3.713 | -3.511 | 0.202 | 0.506 | 0.446 | 0.387 | 0.537 | 0.501 | 0.478 |
| *Chryseobacterium* | -3.506 | -1.953 | 1.858 | -1.552 | -5.366 | -3.811 | 0.498 | 0.334 | 0.349 | 0.476 | 0.501 | 0.399 |
| *Enterovibrio* | 1.520 | -2.415 | 1.077 | 3.934 | 0.443 | -3.492 | 0.732 | 0.316 | 0.362 | 0.718 | 0.747 | 0.392 |
| *Pseudoalteromonas* | -2.431 | -1.959 | -1.099 | -0.472 | -1.332 | -0.860 | 0.609 | 0.388 | 0.366 | 0.631 | 0.621 | 0.424 |
| *Catenococcus* | 2.374 | 0.223 | 0.762 | 2.151 | 1.613 | -0.538 | 0.853 | 0.356 | 0.337 | 0.861 | 0.859 | 0.408 |
| *Rubritalea* | -0.538 | -0.776 | -0.017 | 0.238 | -0.521 | -0.759 | 0.542 | 0.360 | 0.330 | 0.572 | 0.561 | 0.417 |
| **Taxon** | **W** | | | | | | **p-value** | | | | | |
|  | TR-CT | ST-CT | WT-CT | TR-ST | TR-WT | ST-WT | TR-CT | ST-CT | WT-CT | TR-ST | TR-WT | ST-WT |
| *Vibrio* | -3.063 | -2.850 | -2.497 | -0.822 | -1.179 | -0.422 | **0.003** | **0.005** | **0.014** | 0.412 | 0.412 | 0.674 |
| *Pseudarthrobacter* | 0.000 | -8.837 | -12.160 | 7.032 | 9.234 | 2.193 | 1.000 | **<0.001** | **<0.001** | **<0.001** | **<0.001** | **0.030** |
| Marine Methylotrophic Group 3 | -4.434 | -6.537 | 4.486 | -0.121 | -7.460 | -9.136 | **<0.001** | **<0.001** | **<0.001** | 0.904 | **<0.001** | **<0.001** |
| *Trichodesmium* IMS101 | -0.367 | 0.151 | -14.343 | -0.444 | 5.317 | 10.760 | 0.716 | 0.881 | **<0.001** | 0.660 | **<0.001** | **<0.001** |
| Candidatus Megaira | -7.616 | -0.319 | -0.890 | -6.909 | -7.010 | 0.422 | **<0.001** | 0.751 | 0.376 | **<0.001** | **<0.001** | 0.674 |
| *Chryseobacterium* | -7.036 | -5.840 | 5.324 | -3.259 | -10.706 | -9.546 | **<0.001** | **<0.001** | **<0.001** | **0.002** | **<0.001** | **<0.001** |
| *Enterovibrio* | 2.075 | -7.639 | 2.972 | 5.477 | 0.593 | -8.912 | **0.041** | **<0.001** | **0.004** | **<0.001** | 0.555 | **<0.001** |
| *Pseudoalteromonas* | -3.994 | -5.052 | -3.001 | -0.748 | -2.143 | -2.030 | **<0.001** | **<0.001** | **0.003** | 0.456 | **0.034** | **0.044** |
| *Catenococcus* | 2.784 | 0.628 | 2.261 | 2.497 | 1.877 | -1.320 | 1.000 | 1.000 | 1.000 | **0.014** | 0.064 | 0.190 |
| *Rubritalea* | -0.994 | -2.159 | -0.051 | 0.416 | -0.930 | -1.819 | 1.000 | 1.000 | 1.000 | 0.678 | 0.355 | 0.072 |
| **Taxon** | **q-value** | | | | | |  | | | | | |
|  | TR-CT | ST-CT | WT-CT | TR-ST | TR-WT | ST-WT |  |  |  |  |  |  |
| *Vibrio* | **0.023** | **0.039** | 0.094 | 1.000 | 1.000 | 1.000 |  |  |  |  |  |  |
| *Pseudarthrobacter* | 1.000 | **<0.001** | **<0.001** | **<0.001** | **<0.001** | 0.448 |  |  |  |  |  |  |
| Marine Methylotrophic Group 3 | **<0.001** | <**0.001** | **<0.001** | 1.000 | **<0.001** | **<0.001** |  |  |  |  |  |  |
| *Trichodesmium* IMS101 | 1.000 | 1.000 | **<0.001** | 1.000 | **<0.001** | **<0.001** |  |  |  |  |  |  |
| Candidatus Megaira | **<0.001** | 1.000 | 1.000 | **<0.001** | **<0.001** | 1.000 |  |  |  |  |  |  |
| *Chryseobacterium* | **<0.001** | **<0.001** | **<0.001** | **0.027** | **<0.001** | **<0.001** |  |  |  |  |  |  |
| *Enterovibrio* | 0.283 | **<0.001** | **0.031** | **<0.001** | 1.000 | **<0.001** |  |  |  |  |  |  |
| *Pseudoalteromonas* | **0.001** | **<0.001** | **0.022** | 1.000 | 0.536 | 0.657 |  |  |  |  |  |  |
| *Catenococcus* | 1.000 | 1.000 | 1.000 | 0.154 | 1.000 | 1.000 |  |  |  |  |  |  |
| *Rubritalea* | 1.000 | 1.000 | 1.000 | 1.000 | 1.000 | 1.000 |  |  |  |  |  |  |

**Table S5:** Top ten differently abundant bacterial genera detected across the sampling regions (TR = tropical, ST = subtropical and WT = warm temperate) for the stomach microbiome of a range-extending coral reef fish. Regions were performed against the cold temperate region as the reference variable. Significant different p-values and false discovery rate (FDR) corrected p-value (q-value) shown in bold.


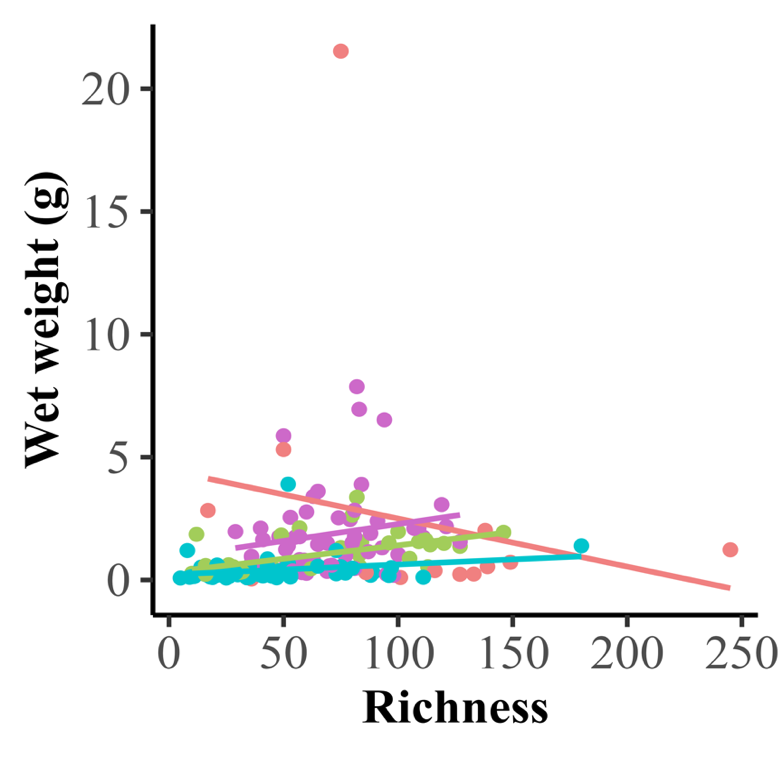

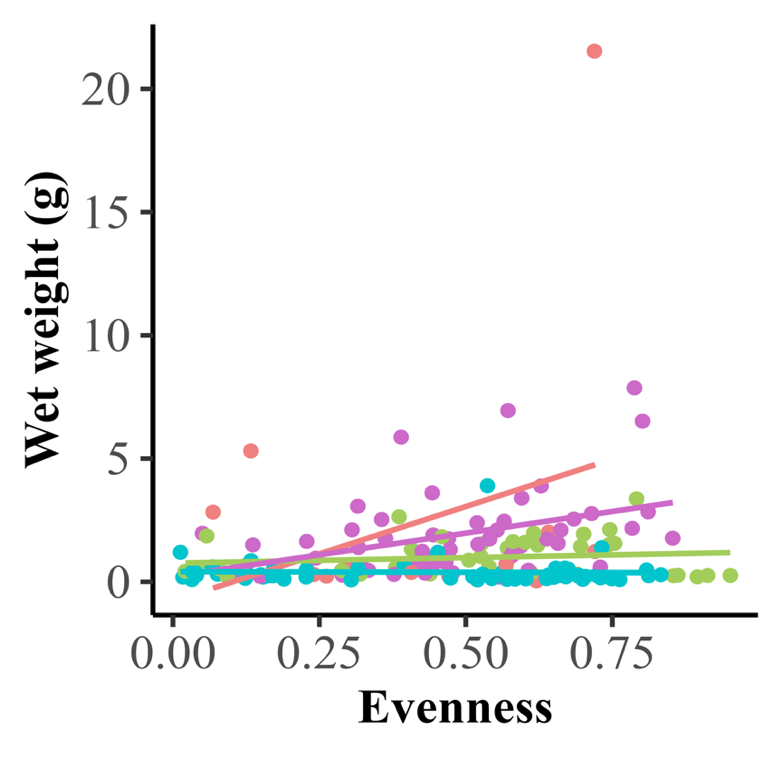

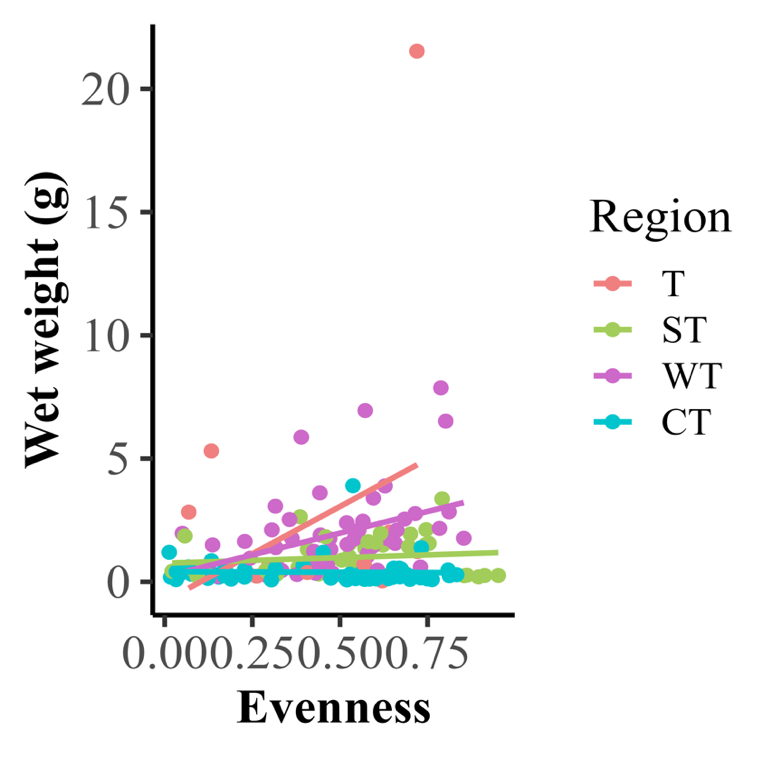


**a**

**b**

**R^2^ = 0.14, p = 0.166**

**R^2^ = 0.06, p = 0.686**

**R^2^ = 0.02, p = 0.469**

**R^2^ = 0.33, p < 0.001***

**R^2^ = 0.16, p = 0.003***

**R^2^ < 0.01, p = 0.847**

**R^2^ = 0.09, p = 0.288**

**R^2^ = 0.02, p = 0.352**

TR

ST

WT

CT

**Figure S3:** Linear regressions showing the relationship between alpha diversity (richness; a, and evenness; b) and wet weight (WW) for a range-extending coral reef fish species across the four study regions. TR represents tropical, ST represents subtropical, WT represents warm temperate, and CT represents cold temperate ranges. Significance is shown by *. See Table S6 for statistical outputs.

**Table S6**: Linear regression outputs for alpha diversity (richness and evenness) and wet weight (WW) of a range-extending coral reef fish species across the four study regions; tropical, subtropical, warm temperate and cold temperate. Significance is shown in bold.

| **Tropical Region** | | | | |
| --- | --- | --- | --- | --- |
| **Richness** | | | | |
| **Coefficients** | **Estimate** | **Std. Error** | **t-value** | **p-value** |
| (Intercept) | 109.087 | 16.354 | 6.670 | **< 0.001** |
| WW | -2.100 | 2.813 | -0.746 | 0.469 |
| Multiple-R^2^: 0.04 | | | | |
| **Evenness** | | | | |
| **Coefficients** | **Estimate** | **Std. Error** | **t-value** | **p-value** |
| (Intercept) | 0.390 | 0.059 | 6.623 | **< 0.001** |
| WW | 0.011 | 0.010 | 1.108 | 0.288 |
| Multiple-R^2^: 0.09 | | | | |
| **Subtropical Region** | | | | |
| **Richness** | | | | |
| **Coefficients** | **Estimate** | **Std. Error** | **t-value** | **p-value** |
| (Intercept) | 32.077 | 8.709 | 3.683 | **< 0.001** |
| WW | 29.760 | 6.943 | 4.286 | **< 0.001** |
| Multiple-R^2^: 0.33 | | | | |
| **Evenness** | | | | |
| **Coefficients** | **Estimate** | **Std. Error** | **t-value** | **p-value** |
| (Intercept) | 0.466 | 0.068 | 6.878 | **< 0.001** |
| WW | 0.051 | 0.054 | 0.943 | 0.352 |
| Multiple-R^2^: 0.02 | | | | |
| **Warm-Temperate Region** | | | | |
| **Richness** | | | | |
| **Coefficients** | **Estimate** | **Std. Error** | **t-value** | **p-value** |
| (Intercept) | 66.607 | 4.767 | 13.971 | **< 0.001** |
| WW | 2.652 | 1.890 | 1.404 | 0.166 |
| Multiple-R^2^: 0.14 | | | | |
| **Evenness** | | | | |
| **Coefficients** | **Estimate** | **Std. Error** | **t-value** | **p-value** |
| (Intercept) | 0.389 | 0.037 | 10.627 | **< 0.001** |
| WW | 0.046 | 0.014 | 3.159 | **0.003** |
| Multiple-R^2^: 0.16 | | | | |
| **Cold-Temperate Region** | | | | |
| **Richness** | | | | |
| **Coefficients** | **Estimate** | **Std. Error** | **t-value** | **p-value** |
| (Intercept) | 36.522 | 4.941 | 7.391 | **< 0.001** |
| WW | 13.842 | 7.456 | 1.857 | 0.686 |
| Multiple-R^2^: 0.06 | | | | |
| **Evenness** | | | | |
| **Coefficients** | **Estimate** | **Std. Error** | **t-value** | **p-value** |
| (Intercept) | 0.461 | 0.043 | 10.662 | **< 0.001** |
| WW | -0.013 | 0.065 | -0.194 | 0.847 |
| Multiple-R^2^: 0.0007 | | | | |


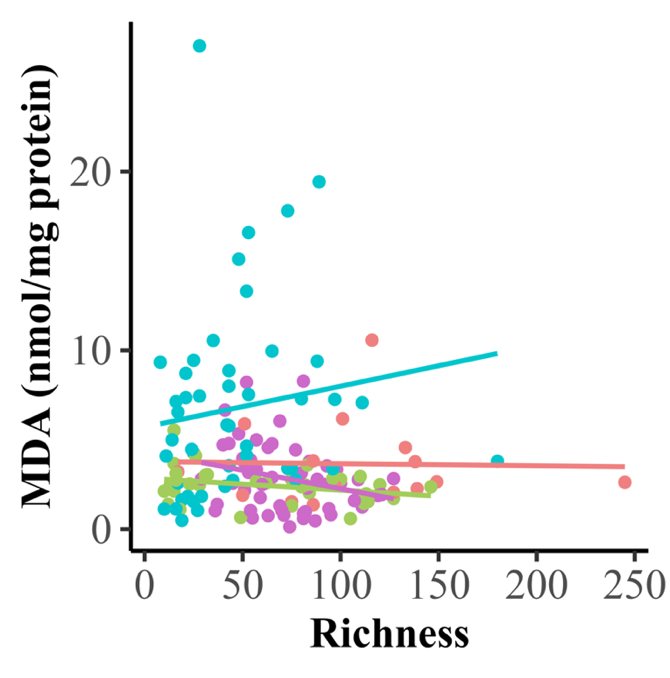

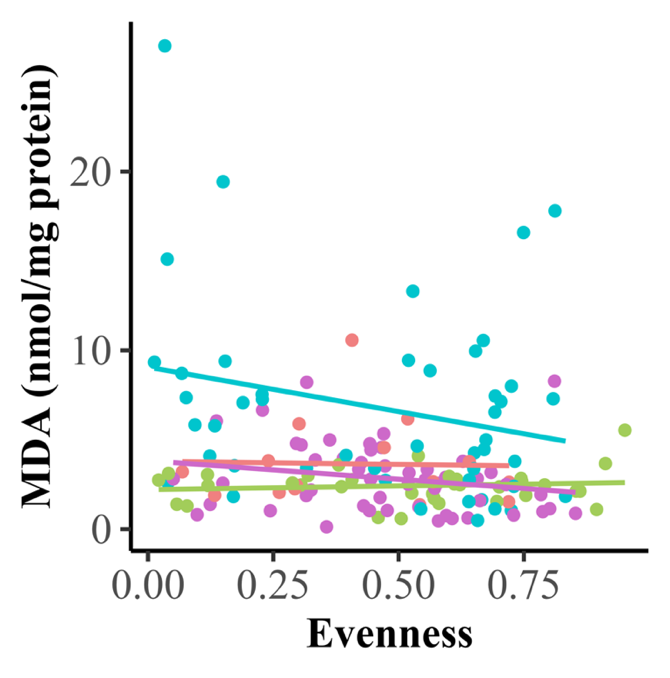

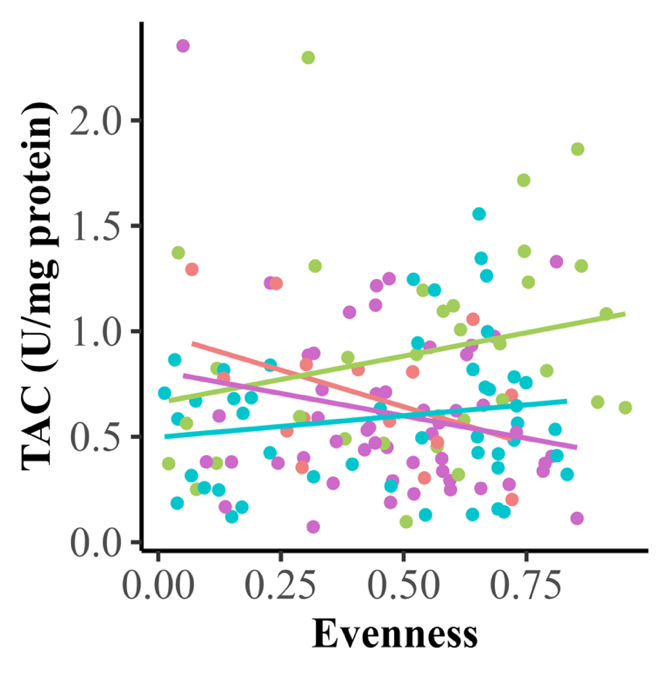

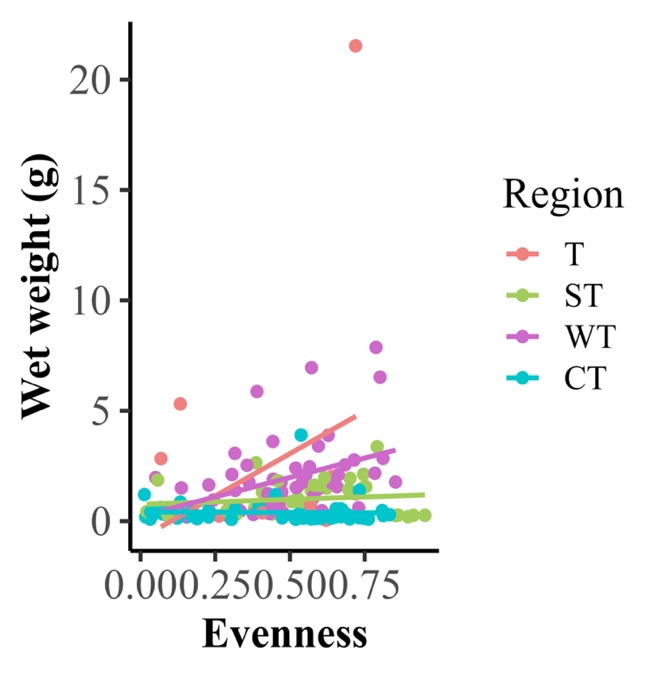

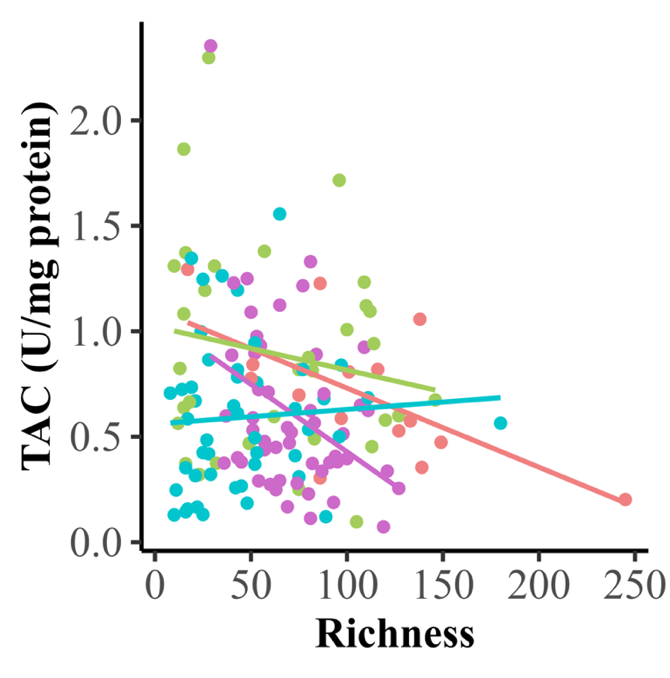


**a**

**b**

**c**

**d**

**R^2^ = 0.07, p = 0.067**

**R^2^ = 0.02, p = 0.347**

**R^2^ = 0.21, p = 0.926**

**R^2^ = 0.09, p = 0.089**

**R^2^ = 0.04, p = 0.135**

**R^2^ = 0.06, p = 0.094**

**R^2^ < 0.01, p = 0.917**

**R^2^ = 0.01, p = 0.511**

**R^2^ = 0.14, p = 0.006***

**R^2^ < 0.01, p = 0.659**

**R^2^ < 0.01, p = 0.012***

**R^2^ = 0.03, p = 0.307**

**R^2^ = 0.04, p = 0.149**

**R^2^ = 0.02, p = 0.289**

**R^2^ = 0.20, p = 0.098**

**R^2^ = 0.06, p = 0.156**

TR

ST

WT

CT

**Figure S4:** Linear regressions showing the relationship between alpha diversity (richness and evenness) and cellular defence (TAC) and cellular stress (MDA) of a range-extending coral reef fish across the four locations (TR = tropical, ST = subtropical, WT = warm temperate, CT = cold temperate).

**Table S7**: Linear regression outputs for oxidative stress (TAC and MDA) and alpha diversity measure richness of a range-extending coral reef fish species across the four study regions; tropical, subtropical, warm temperate and cold temperate. Significance is shown in bold.

| **Tropical Region** | | | | |
| --- | --- | --- | --- | --- |
| **Cellular defence (TAC)** | | | | |
| **Coefficients** | **Estimate** | **Std. Error** | **t-value** | **p-value** |
| (Intercept) | 181.33 | 27.78 | 6.527 | **< 0.001** |
| TAC | -105.26 | 36.16 | -2.911 | **0.012** |
| Multiple-R^2^: 0.0007 | | | | |
| **Cellular damage (MDA)** | | | | |
| **Coefficients** | **Estimate** | **Std. Error** | **t-value** | **p-value** |
| (Intercept) | 109.4817 | 26.8230 | 4.082 | **0.001** |
| MDA | 109.4817 | 6.1920 | -0.095 | 0.926 |
| Multiple-R^2^: 0.21 | | | | |
| **Subtropical Region** | | | | |
| **Cellular defence (TAC)** | | | | |
| **Coefficients** | **Estimate** | **Std. Error** | **t-value** | **p-value** |
| (Intercept) | 77.89 | 15.41 | 5.055 | **< 0.001** |
| TAC | -15.83 | 15.24 | -1.038 | 0.307 |
| Multiple-R^2^: 0.03 | | | | |
| **Cellular damage (MDA)** | | | | |
| **Coefficients** | **Estimate** | **Std. Error** | **t-value** | **p-value** |
| (Intercept) | 95.266 | 19.274 | 4.943 | **< 0.001** |
| MDA | -12.976 | 7.392 | -1.755 | 0.089 |
| Multiple-R^2^: 0.09 | | | | |
| **Warm-Temperate Region** | | | | |
| **Cellular defence (TAC)** | | | | |
| **Coefficients** | **Estimate** | **Std. Error** | **t-value** | **p-value** |
| (Intercept) | 85.584 | 5.703 | 15.007 | **< 0.001** |
| TAC | -22.406 | 7.826 | -2.863 | **0.006** |
| Multiple-R^2^: 0.14 | | | | |
| **Cellular damage (MDA)** | | | | |
| **Coefficients** | **Estimate** | **Std. Error** | **t-value** | **p-value** |
| (Intercept) | 81.160 | 5.900 | 13.756 | **< 0.001** |
| MDA | -3.239 | 1.727 | -1.876 | 0.067 |
| Multiple-R^2^: 0.07 | | | | |
| **Cold-Temperate Region** | | | | |
| **Cellular defence (TAC)** | | | | |
| **Coefficients** | **Estimate** | **Std. Error** | **t-value** | **p-value** |
| (Intercept) | 42.469 | 9.714 | 4.372 | **< 0.001** |
| TAC | 6.285 | 14.164 | 0.444 | 0.659 |
| Multiple-R^2^: 0.004 | | | | |
| **Cellular damage (MDA)** | | | | |
| **Coefficients** | **Estimate** | **Std. Error** | **t-value** | **p-value** |
| (Intercept) | 40.3690 | 7.8188 | 5.163 | **< 0.001** |
| MDA | 0.8606 | 0.9054 | 0.951 | 0.347 |
| Multiple-R^2^: 0.02 | | | | |

**Table S8**: Linear regression outputs for oxidative stress (TAC and MDA) and alpha diversity measure evenness of a range-extending coral reef fish species across the four study regions; tropical, subtropical, warm temperate and cold temperate. Significance is shown in bold.

| **Tropical Region** | | | | |
| --- | --- | --- | --- | --- |
| **Cellular defence (TAC)** | | | | |
| **Coefficients** | **Estimate** | **Std. Error** | **t-value** | **p-value** |
| (Intercept) | 0.612 | 0.122 | 5.016 | **< 0.001** |
| TAC | -0.283 | 0.159 | -1.783 | 0.098 |
| Multiple-R^2^: 0.20 | | | | |
| **Cellular damage (MDA)** | | | | |
| **Coefficients** | **Estimate** | **Std. Error** | **t-value** | **p-value** |
| (Intercept) | 0.421 | 0.102 | 4.129 | **< 0.001** |
| MDA | -0.003 | 0.024 | -0.107 | 0.917 |
| Multiple-R^2^: 0.0009 | | | | |
| **Subtropical Region** | | | | |
| **Cellular defence (TAC)** | | | | |
| **Coefficients** | **Estimate** | **Std. Error** | **t-value** | **p-value** |
| (Intercept) | 0.394 | 0.097 | 4.056 | **< 0.001** |
| TAC | 0.140 | 0.096 | 1.452 | 0.156 |
| Multiple-R^2^: 0.06 | | | | |
| **Cellular damage (MDA)** | | | | |
| **Coefficients** | **Estimate** | **Std. Error** | **t-value** | **p-value** |
| (Intercept) | 0.439 | 0.129 | 3.422 | **< 0.001** |
| MDA | 0.033 | 0.049 | 0.664 | 0.511 |
| Multiple-R^2^: 0.01 | | | | |
| **Warm-Temperate Region** | | | | |
| **Cellular defence (TAC)** | | | | |
| **Coefficients** | **Estimate** | **Std. Error** | **t-value** | **p-value** |
| (Intercept) | 0.539 | 0.049 | 10.950 | **< 0.001** |
| TAC | -0.099 | 0.068 | -1.467 | 0.149 |
| Multiple-R^2^: 0.04 | | | | |
| **Cellular damage (MDA)** | | | | |
| **Coefficients** | **Estimate** | **Std. Error** | **t-value** | **p-value** |
| (Intercept) | 0.540 | 0.049 | 11.088 | **< 0.001** |
| MDA | -0.217 | 0.014 | -1.519 | 0.135 |
| Multiple-R^2^: 0.04 | | | | |
| **Cold-Temperate Region** | | | | |
| **Cellular defence (TAC)** | | | | |
| **Coefficients** | **Estimate** | **Std. Error** | **t-value** | **p-value** |
| (Intercept) | 0.390 | 0.078 | 4.995 | **< 0.001** |
| TAC | 0.122 | 0.113 | 1.074 | 0.289 |
| Multiple-R^2^: 0.02 | | | | |
| **Cellular damage (MDA)** | | | | |
| **Coefficients** | **Estimate** | **Std. Error** | **t-value** | **p-value** |
| (Intercept) | 0.545 | 0.062 | 8.782 | **< 0.001** |
| MDA | -0.012 | 0.007 | -1.713 | 0.094 |
| Multiple-R^2^: 0.06 | | | | |


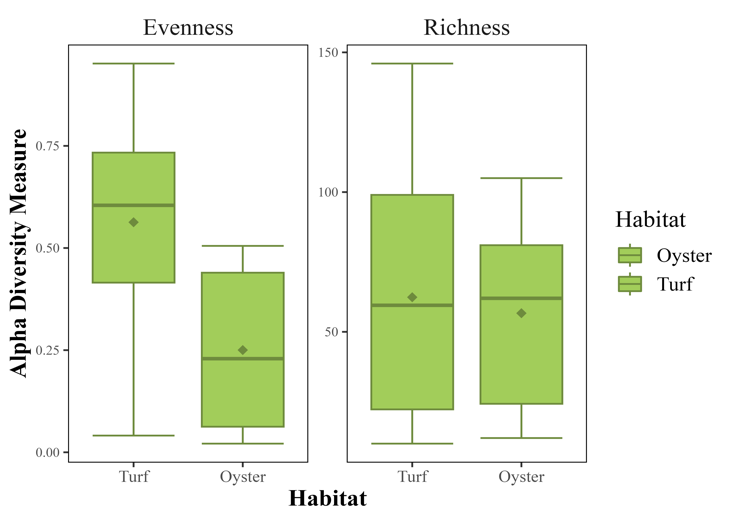

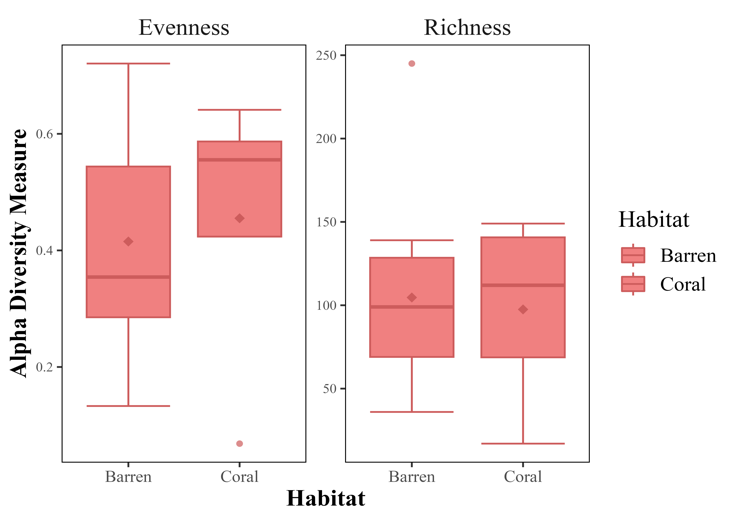

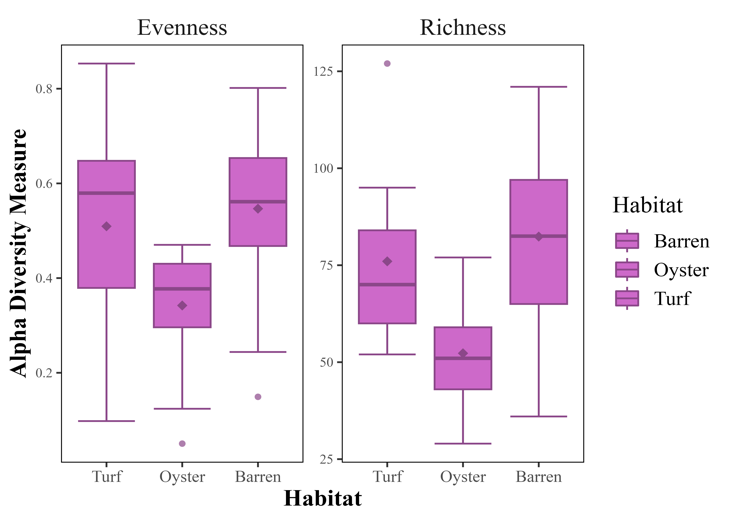

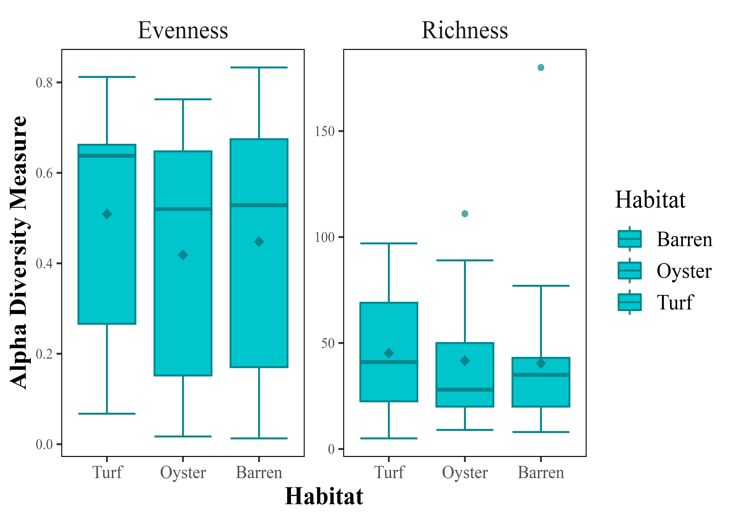

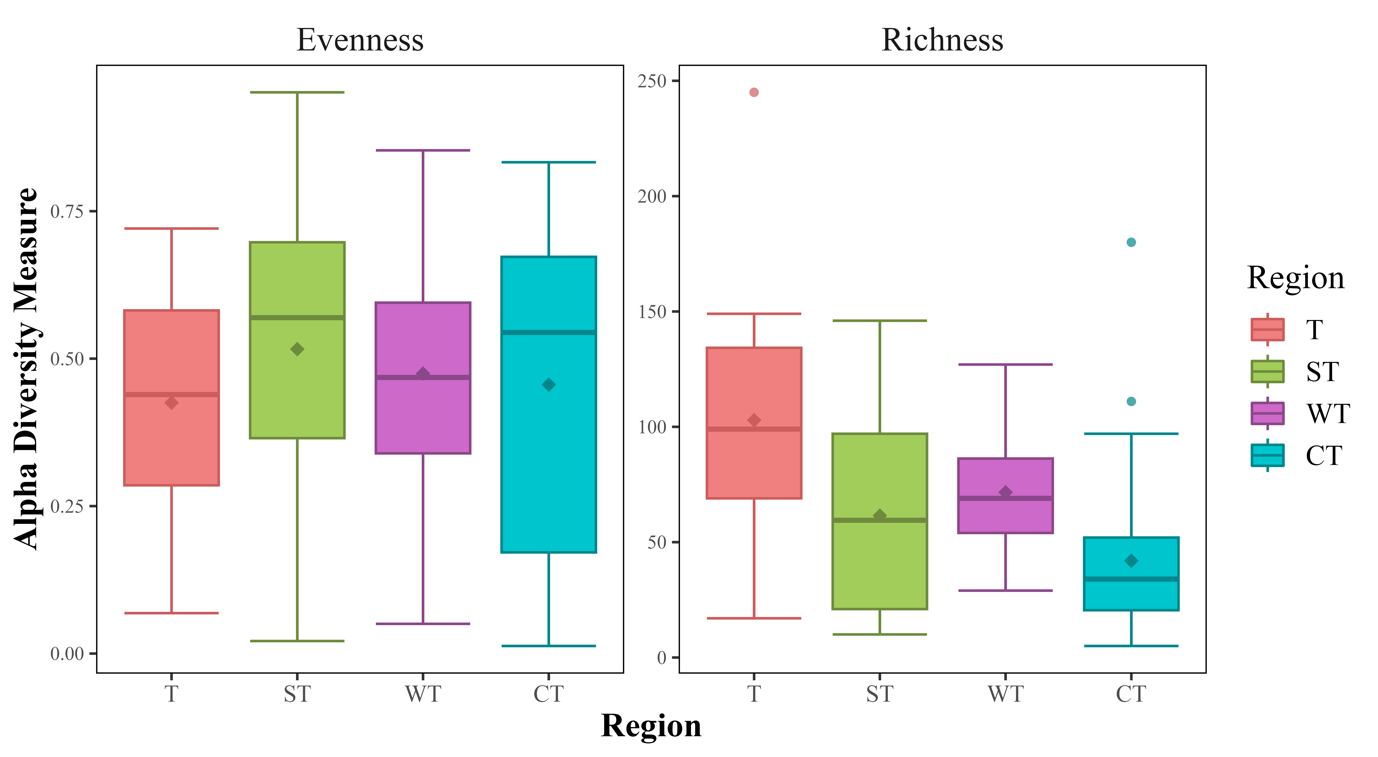


**KS: < 0.001**

**NS**

**NS**

**NS**

**NS**

**NS**

**A**

**B**

**B**

**A**

**A**

**A**

**A**

**B**

**a**

**b**

**c**

**d**

**KS: < 0.001**

**KS: 0.006**

TR

ST

WT

CT

**Figure S5:** Boxplots showing differences in alpha diversity measures evenness and richness of *Abudefduf vaigiensis* stomach microbiome across the different climatic regions (TR = tropical, ST = subtropical, WT = warm temperate, CT = cold temperate). The boxes represent the lower and upper quartile, whiskers represent the extremum values, horizonal lines show the median and diamonds show the mean. KS indicates the degree of significance for the main test (Kruskal-Wallis test) and the different letters indicate the Dunn’s *post hoc* test significant differences between regions (p < 0.05).

**Table S9:** Kruskal-Wallis and Dunn-tests for alpha diversity measures richness and evenness for each habitat type present at the sampling regions. Significant p-values shown in bold (<0.05).

| **Kruskal-Wallis (richness)** | | | | | | |
| --- | --- | --- | --- | --- | --- | --- |
| Region | | Kruskal-Wallis χ^2^ | | df | p-value | |
| Tropical | | 0.033 | | 1 | 0.856 | |
| Subtropical | | 0.224 | | 1 | 0.636 | |
| Warm Temperate | | 19.453 | | 2 | **<0.001** | |
| Cold Temperate | | 0.293 | | 2 | 0.867 | |
| **Post-hoc Dunn-test (richness)** | | | | | | |
| Region | Habitat | | Z | | p-value |  |
| Warm Temperate  Warm Temperate  Warm Temperate | Barren-Oyster | | -4.35 | | **< 0.001** |  |
|  | Barren-Turf | | -0.78 | | 0.433 |  |
|  | Oyster-Turf | | 2.77 | | **0.006** |  |
| **Kruskal-Wallis (evenness)** | | | | | | |
| Region | | Kruskal-Wallis χ^2^ | | df | p-value | |
| Tropical | | 0.235 | | 1 | 0.628 | |
| Subtropical | | 7.646 | | 1 | **0.006** | |
| Warm Temperate | | 14.978 | | 2 | **<0.001** | |
| Cold Temperate | | 0.724 | | 2 | 0.696 | |
| **Post-hoc Dunn-test (evenness)** | | | | | | |
| Region | Habitat | | Z | | p-value |  |
| Warm Temperate  Warm Temperate  Warm Temperate | Barren-Oyster | | -3.76 | | **< 0.001** |  |
|  | Barren-Turf | | -0.43 | | 0.666 |  |
|  | Oyster-Turf | | 2.63 | | **0.009** |  |
| **Post-hoc Dunn-test (evenness)** | | | | | | |
| Region | Habitat | | Z | | p-value |  |
| Subtropical | Oyster-Turf | | 2.77 | | **0.006** |  |


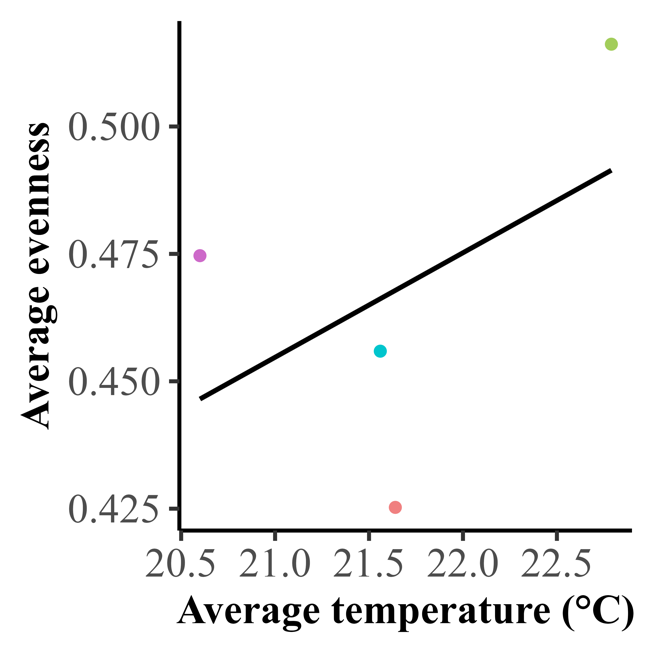

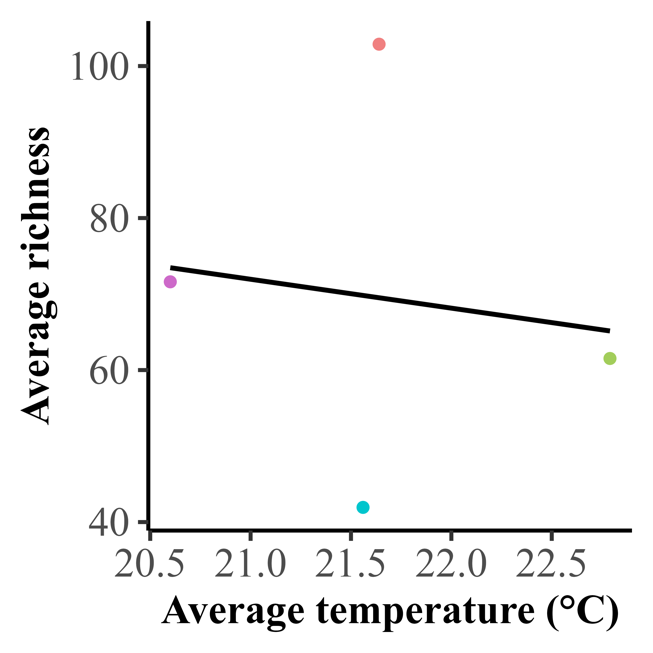

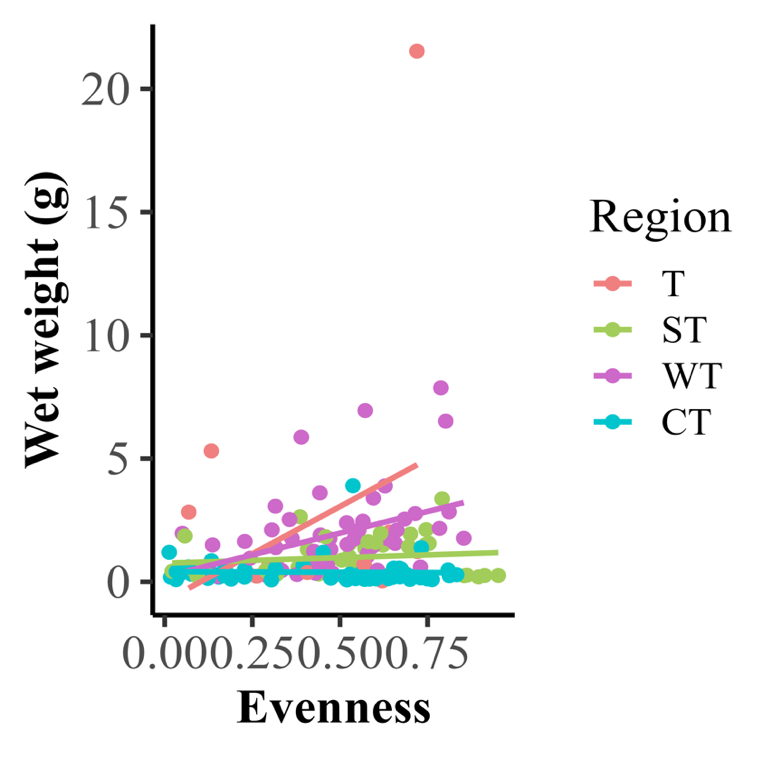


TR

WT

CT

ST

**R^2^ = 0.02, p = 0.866**

**b**

**a**

**R^2^ = 0.23, p = 0.517**

**Figure S6:** Linear regressions showing the relationship between alpha diversity (richness; a, and evenness; b) and average water temperature (°C) for the four study regions. TR represents tropical, ST represents subtropical, WT represents warm temperate, and CT represents cold temperate ranges. See Table S10 for statistical outputs.

**Table S10**: Linear regression outputs for alpha diversity (richness and evenness) and average water temperature of a range-extending coral reef fish species across the four study regions; tropical, subtropical, warm temperate and cold temperate. Significance is shown in bold.

| **Richness** | | | | |
| --- | --- | --- | --- | --- |
| **Coefficients** | **Estimate** | **Std. Error** | **t-value** | **p-value** |
| (Intercept) | 21.975 | 1.800 | 12.211 | **0.006** |
| Temperature | -0.005 | 0.025 | 0.025 | 0.866 |
| Multiple-R^2^: 0.02 | | | | |
| **Evenness** | | | | |
| **Coefficients** | **Estimate** | **Std. Error** | **t-value** | **p-value** |
| (Intercept) | 16.313 | 6.847 | 2.383 | 0.140 |
| Temperature | 11.398 | 14.594 | 14.594 | 0.517 |
| Multiple-R^2^: 0.23 | | | | |
